# Integration and scaling of UV-B radiation effects on plants: from DNA to leaf

**DOI:** 10.1002/ece3.1332

**Published:** 2015-06-02

**Authors:** Vasile Alexandru Suchar, Ronald Robberecht

**Affiliations:** Department of Forest, Rangeland, and Fire Sciences, College of Natural Resources, University of Idaho875 Perimeter Drive MS1133, Moscow, Idaho, 83844-1133

**Keywords:** DNA damage, ecological integration, feedbacks, leaf area, ozone depletion, phenolics, UV-B radiation

## Abstract

A process-based model integrating the effects of UV-B radiation through epidermis, cellular DNA, and its consequences to the leaf expansion was developed from key parameters in the published literature. Enhanced UV-B radiation-induced DNA damage significantly delayed cell division, resulting in significant reductions in leaf growth and development. Ambient UV-B radiation-induced DNA damage significantly reduced the leaf growth of species with high relative epidermal absorbance at longer wavelengths and average/low pyrimidine cyclobutane dimers (CPD) photorepair rates. Leaf expansion was highly dependent on the number of CPD present in the DNA, as a result of UV-B radiation dose, quantitative and qualitative absorptive properties of epidermal pigments, and repair mechanisms. Formation of pyrimidine-pyrimidone (6-4) photoproducts (6-4PP) has no effect on the leaf expansion. Repair mechanisms could not solely prevent the UV-B radiation interference with the cell division. Avoidance or effective shielding by increased or modified qualitative epidermal absorptance was required. Sustained increased UV-B radiation levels are more detrimental than short, high doses of UV-B radiation. The combination of low temperature and increased UV-B radiation was more significant in the level of UV-B radiation-induced damage than UV-B radiation alone. Slow-growing leaves were more affected by increased UV-B radiation than fast-growing leaves.

## Introduction

Ultraviolet (UV) radiation has been a natural environmental stress factor for organisms since the pre-Cambrian era (Lowry et al. 1980; Rettberg et al. 1998; Cockell and Horneck 2001). Ultraviolet radiation induces injury to DNA, causes DNA mutations, inhibits photosynthetic processes, impairs membrane function, and can cause lethal cell damage (Sancar and Sancar 1988; Britt 1996; Taylor et al. 1997; Rozema et al. 1999; Weber 2005). In addition to such direct UV-induced damage, DNA mutations may have been the catalysts for phylogenetic diversity through accelerated selection and evolution (Sagan 1973; Cockell 2000) and, as a result, be, at least partly, responsible for the success of terrestrial plant species (Lowry et al. 1980; Stafford 1991; Rozema et al. 1999).

Current stratospheric ozone depletion and the potential-associated UV-B radiation increase can significantly affect terrestrial plant species (Searles et al. 2001; Day and Neale 2002), and these changes may be amplified across higher ecological scales and trophic levels (Caldwell et al. 1998; van der Leun et al., 1998, Warren et al. 2002). Furthermore, stratospheric ozone depletion and global warming may be producing significant changes in both surface and stratospheric climate (Hartmann et al. 2000; United Nations Environment Programme EEAP 2012). Thus, understanding how different levels of UV radiation environment of Earth affect terrestrial communities is important in predicting how the current stratospheric ozone depletion may affect life on Earth and may interact with climate changes toward rapid global change. Also, it may provide insights into the UV radiation contribution as a selection agent throughout the evolutionary history of Earth.

Yet, experimental research on UV radiation effects on organisms has been mostly limited to individual and subindividual plant levels. This is largely due to the technical difficulties in simulating an enhanced UV-B radiation regime at the scales required for higher ecological-level experiments (DeLucia et al. 2001). A modeling research approach, which integrates and scales the effects of enhanced UV-B radiation on terrestrial plant communities, was therefore used to understand plant response mechanisms to UV-B radiation and their broader consequences, identify the processes insufficiently addressed by past research, as well as to investigate hypotheses that were untestable by experimental research.

We modeled the pathway of UV-B radiation in leaf, its qualitative and quantitative attenuation in epidermis, its effects upon plant DNA, cellular responses to DNA injury, and their potential consequences on leaf growth and development. Our primary hypothesis was that enhanced solar UV-B radiation-induced DNA damage significantly reduces leaf growth and development. Damage to DNA above ambient levels might delay cell division until the injury is repaired, or might delay cell expansion (Srivastava 2002; Lo et al. 2005; De Lima-Bessa et al. 2008; Hectors et al. 2010). Delays in cell division and expansion during leaf expansion and possible cell apoptosis might lead to modifications in leaf morphology, such as decreased leaf size, or even premature leaf senescence. These processes can significantly reduce the photosynthetic capacity of leaves, with consequences upon whole plant growth and development (Rozema et al. 1997; Caldwell et al. 1998; Milchunas et al. 2004).

Although there is considerable research regarding the effects of UV-B radiation on concentration of flavonoids and related phenolics compounds, UV-B induced DNA injuries, and the effects of UV-B radiation on leaf morphology, our model integrated these processes and showed how changes in molecular and cellular processes can result in whole organ changes. We were able to examine a variety of questions that were difficult to approach through experimental research, including the following: (1) Are long, sustained increased UV-B radiation levels more detrimental than short, high doses of UV-B radiation? (2) Are fast-growing leaves more adaptive than slow-growing leaves? (3) Are different relative absorption spectra of flavonoids and related phenolics compounds responsible for the observed physiology of the leaf? (4) How important is DNA repair in leaf development? and (5) Is there an interaction between temperature and UV-B radiation-induced effects?

## Model Framework

The model is comprised of four major components: UV-B radiation, leaf optical properties, DNA damage and repair mechanisms, and leaf cell division and expansion. The dose rate of UV-B radiation is a fundamental component of the model, as it influences the quantity of epidermal pigments, their absorption spectra, and the quantity and quality of damaging radiation reaching the DNA.

Once incident on the leaf, the UV-B radiation pathway into the leaf is determined by the leaf optical properties (reflectance, absorptance, and transmittance). Most plant species exhibit low levels of UV-B leaf surface reflectance (5–6%), although some species can reflect up to 70% (Gausman et al. 1975; Robberecht and Caldwell 1978; Robberecht et al. 1980). Generally, 85–95% of the UV-B radiation is absorbed by the leaf, and the remaining UV-B radiation is transmitted (Gausman et al. 1975; Robberecht and Caldwell 1978; Robberecht et al. 1980; Bieza and Lois 2001). Pigments, primarily flavonoids, isoflavonoids, sinapate esters, flavons, and anthocyanins, are the most important leaf constituents that absorb UV-B radiation (Robberecht and Caldwell 1978; Robberecht et al. 1980; Koes et al. 1994; Dixon and Paiva 1995; Winkel-Shirley 2002). Increases in UV-B radiation generally stimulate the production of secondary metabolites and result in changes in epidermal absorption (Schmelzer et al. 1988; Li et al. 1993; Koes et al. 1994; Dixon and Paiva 1995; Winkel-Shirley 2002). The relative changes in the quantity and quality of secondary metabolites vary with species (Li et al. 1993; Dixon and Paiva 1995; Chalker-Scott 1999). Regardless of the compounds and amounts produced, their relative absorption spectra follow three general patterns (Sisson 1981; Schmelzer et al. 1988; Day et al. 1994; Lavola et al. 1997; Qi et al. 2003). Most evergreen species, deciduous trees, shrubs, and vines show a maximum absorption at shorter wavelength (280 nm) and lower relative absorption at longer UV-B radiation wavelengths. Most grasses and herbaceous plants show minimum absorption at shorter wavelengths and greater relative absorption at longer UV-B radiation wavelengths.

The major DNA lesions induced by UV-B radiation include pyrimidine cyclobutane dimers (CPD) and pyrimidine-pyrimidone (6-4) photoproducts (6-4PP) (Sancar and Sancar 1988; Britt 1996; Taylor et al. 1997; Weber 2005). Low UV-B radiation doses induce CPD to 6-4PP ratio of approximately 9:1, while very high UV-B radiation doses result in 6:4 ratios (Sancar 2003). Photoproducts are reversed through photorepair and nucleotide excision repair (NER) or dark repair. The CPD photolyase and 6-4PP photolyase bind to the DNA injury and reverse the damage using 350–450 nm light as energy source (Sancar 2003; Weber 2005). The 6-4PP photorepair is more efficient than CPD photorepair (Chen et al. 1994; Jiang et al. 1997). But, the CPD photolyase quantum yields are higher than those of 6-4 photolyase (Sancar 2003). Nucleotide excision repair (NER) is an ATP-dependent, complex repair pathway, involved in the removal of a variety of bulky DNA lesions including CPDs and 6-4PPs. NER repair of 6-4PPs is 9.5–10.7 faster than NER repair of CPDs (Sancar 2003; Lo et al. 2005; De Lima-Bessa et al. 2008).

Induction and repair mechanisms rates are temperature dependent. Photoproducts induction rates at 0°C are the lowest, increase with temperature, and stabilize or decline above 30°C (Takeuchi et al. 1996; Li et al. 2002; Waterworth et al. 2002). The photoproducts repair rates are also temperature dependent: negligible at 0°C, increase with temperature, and remain steady or decline above 30°C (Takeuchi et al. 1996; Li et al. 2002; Waterworth et al. 2002). But, the potential accumulation of photoproducts in plants growing in low-temperature environments as a result of low rate of UV-B radiation photoproducts induction and negligible repair rates might be mediated by a low-temperature stimulation of screening compounds production (Bilger et al. 2007).

Small unrepaired CPDs and 6-4PPs numbers arrest the cell cycle to allow effective repair, while major damage can induce apoptosis (Lo et al. 2005). Unrepaired 6-4PPs trigger apoptosis, whereas unrepaired CPDs rather induce cell cycle arrest (Lo et al. 2005). In NER-deficient cells, both CPDs and 6-4PPs lead to apoptosis, while in NER-proficient cells, CPDs were solely responsible for apoptosis as 6-4PPs were rapidly repaired by NER (De Lima-Bessa et al. 2008). However, either DNA lesions, if unrepaired after 24 h, lead to apoptosis at noncumulative rates (Lo et al. 2005; De Lima-Bessa et al. 2008). Apoptosis triggering by UV-B radiation-induced lesions is delayed minimum 8-16 h, probably to allow time for damage removal (Lo et al. 2005; De Lima-Bessa et al. 2008). While these results were recorded for human cells, it is plausible that similar mechanisms may also regulate plant cells life cycles.

Enhanced UV-B radiation has been shown to induce smaller leaves in many plant species (Teramura et al. 1991; González et al. 1998), as a result of decreased leaf growth rates mainly during the day period (Hopkins et al. 2002). The leaf growth process is driven initially by active cell division, followed by cell expansion and differentiation, and leaf maturity (Beemster et al. 2005). Ultraviolet radiation may inhibit cell division (González et al. 1998; Rousseaux et al. 2004), cell expansion (Wargent et al. 2009b; Hectors et al. 2010), or both (Hopkins et al. 2002; Hofmann et al. 2003; Wargent et al. 2009b). While the connection between UV-B radiation, induction of DNA damage, and cell cycle arrest or apoptosis seems clear (Britt 1996; Lo et al. 2005; Weber 2005; De Lima-Bessa et al. 2008), the mechanisms of UV-B radiation-induced reduced cell expansion rates are less understood (Hectors et al. 2010). While DNA is a key receptor of UV-B radiation, several plant stress signaling components (e.g., NADPH oxidase-derived reactive oxygen species, jasmonic acid, nitric oxide, mitogen-activated protein kinases) may be affected by enhanced UV-B radiation, with possible inhibitory effects on leaf expansion (Wargent et al. 2009a; Ballare et al. 2011). Ultraviolet-B-specific signaling proteins (e.g., as UVR8) have been shown to regulate gene activity responsible for secondary metabolites production and photorepair of DNA lesions (Brown et al. 2005; Ballare et al. 2011). For example, in *Arabidopsis thaliana*, UVR8 is involved in leaf growth and photomorphogenesis by controlling leaf cell expansion, but it has no effect on cell division (Wargent et al. 2009a). Primary literature presenting these effects were discussed in previous papers (Wargent et al. 2009a,b; Ballare et al. 2011).

Our research modeled these processes for a hypothetical generalized leaf (a simple, planophyllic, glabrous, green plant leaf) and integrated the effects of UV-B radiation on DNA and the consequences on the leaf expansion over one growing season. This generalized leaf allowed us to model the influence of UV-B radiation under a variety of scenarios, including variations in leaf characteristics, UV-B irradiance, and repair mechanisms.

## Model Architecture

**Figure 1:**
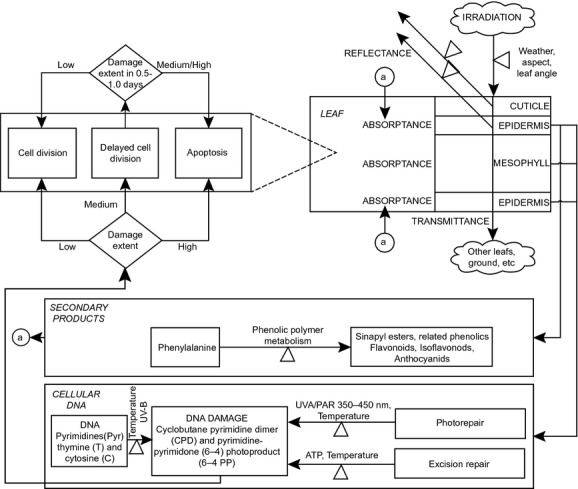
UV-B radiation is reflected, absorbed, or transmitted through the leaf. Most of the absorbed UV-B radiation is retained by epidermal secondary metabolites. Production of secondary metabolites is stimulated by increased UV-B radiation. Remaining absorbed UV-B radiation induces DNA injuries, which are repaired through UVA/PAR or ATP-catalyzed repair mechanisms, or targets signaling proteins with photomorphogenic effects on cell expansion. Medium to high DNA injuries levels can arrest cell cycle or trigger apoptosis. Acronyms used in the figure: Pyrimidines (Pyr), Thymine (T), Cytosine (C), Cyclobutane pyrimidine dimmers (CPDs), Pyrimidine-pyrimidone (6-4) photoproducts (6-4PPs), Ultraviolet-A radiation (UVA), Photosynthetically Active Region (PAR), Adenosine Triphosphate (ATP).

Our model simulated the leaf optical properties (reflectance, absorptance, and transmittance) under various levels of UV-B irradiation, the absorptance of epidermal secondary metabolites, the UV-B radiation targeting of signaling proteins and their photomorphogenic effects on cell expansion, the UV-B radiation induction of DNA injuries, their repair through UVA/PAR or ATP-catalyzed repair mechanisms, their consequences on leaf cell cycle, and leaf expansion (Fig.1). A complete presentation of the mathematical model and parameters estimation is presented in the supporting information file.

The model was created in Vensim modeling software (Systems 2009). Data compilation, preparation, and analysis were performed in various programs such as Microsoft Access, Excel, and R-language (Team RDC 2010).

The models were verified for consistency and units, for correctness of the mathematics, and for accuracy of the conceptual logic (Rykiel 1996), and calibrated and validated (Shugart 1984; Rykiel 1996; Gardner and Urban 2003). Prior to this, sensitivity analysis procedure was performed (Plentinger and Penning De Vries 1996; Rykiel 1996; Aber et al. 2003).

## Model Analysis

### Sensitivity analysis

The ranges derived for the major model parameters were used for the allowable limits used in the model sensitivity analysis and calibration. The following parameters were tested: leaf optical properties (*k*_*R*_, and *k*_*T*_), CPD induction (*k*_*A,DNA,CPD*_ and *k*_*c*_), CPD/6-4PP photorepair and dark repair (*a* and *r*_*max*_), CPD/6-4PP levels over which cell division is delayed (*k*_*cdd*_), and duration of the cell division delay (*t*_*cdd*_). The relative maximum number of CPDs and 6-4PPs during the leaf growing period and the relative mature leaf area were measured across the tested model parameters (Fig. 7).

Our results show that the number of CPDs during the leaf growing period is sensitive to the amount of UV-B radiation reaching the DNA, the rate of CPD induction, and CPD photorepair and dark repair rate multipliers. The number of 6-4PPs during the leaf growing period is sensitive to the amount of UV-B radiation reaching the DNA, the rate of 6-4PPs induction, and 6-4PP photorepair and dark repair rate multipliers. The CPD and 6-4PP in DNA do not reach the levels corresponding to the maximum CPDs and 6-4PPs photorepair and dark repair rates, for either UV-B radiation dose. Leaf area is sensible only to changes in CPDs levels. The 6-4PPs do not reach any levels that can influence leaf growth and expansion. Also, it is sensible to the CPD level at which cell division is delayed.

DNA lesions induction rate is the most influential factor, and it is responsible for the highest variation in CPDs and 6-4PPs numbers, and relative leaf area. Repair of CPDs is less influential on the model, while the model is resistant to changes in 6-4PPs concentrations.

### Calibration and validation

Because the diversity of experiments used to infer the parameter values prevented a species-specific calibration and validation, the model was calibrated by trial-and-error adjustment of the most sensitive parameters. Data collected for rice species were used for the evaluation of the CPD induction and repair rates (Quaite et al. 1994; Kang et al. 1998; Hidema et al. 2001, 2007; Iwamatsu et al. 2008). Field-grown rice showed concentrations of 3-6 CPDs Mb^−1^ during the day, if grown under ambient UV-B conditions and higher values under UV-B supplementation (Hidema et al. 2000, 2001). Moreover, rice cultivars seem to show decreases of about 50% decrease in dry weight under increased UV-B radiation exposure (Hidema et al. 2001; Iwamatsu et al. 2008), depending on the effectiveness of their CPD repair mechanisms. For the calibration purposes, we considered these values to be equivalent to roughly 50% decrease in leaf area.

As validation, we considered that the model should show the general trends observed in previous experiments. First, leaves of many tree species do not have significantly smaller leaf size at higher UV-B radiation doses. Second, different combinations of repair rates should result in decreases about 20–90% dry weight (or approximate 20–90% leaf area) when plants are exposed to no UV-B and to 3.6 KJ m^−2 ^h^−1^ (Iwamatsu et al. 2008).

The parameter estimates following the model calibration are presented in Table1. For the CPD/6-4PP levels over which cell division is delayed, we choose, instead of a singular value, a range of 5 to 14 CPD/6-4PP. For CPD/6-4PP values smaller or equal than 5 CPD/6-4PP, the cell division is not delayed; for values above 14 CPD/6-4PP, cell division is 100 percent delayed; and for values between 5 and 14 CPD/6-4PP, cell division is proportionally delayed. The value of the supplemental UV-B radiation absorbed by epidermal pigments, when exposed to increased UV-B radiation (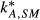), was more difficult to estimate. The range of the values was inferred from a range of experimental designs (Tevini et al. 1981, 1982, 1983; Li et al. 1993; Liu et al. 1995; Vandestaaij et al. 1995; Day and Demchik 1996; Sheahan 1996; Bornman et al. 1997; Olsson et al. 1998; Meijkamp et al. 1999; De La Rosa et al. 2001; Kolb et al. 2001; Tegelberg et al. 2003). Epidermal pigment content was compared between plants grown with no UV-B radiation exposure and under various UV-B radiation doses. Moreover, solar UV-B radiation might have a greater influence on the epidermal pigments content than the increased UV-B radiation (Ryan et al. 1998, 2002). As the range is too wide, study conditions were too diverse, and extrapolation of rates from one range of UV-B radiation doses to a different one is problematic, we considered for this model that the epidermal UV-B absorptance is constant (0.94) for any level of UV-B irradiance. We recognize that this value might lead to imprecise model predictions especially at increased UV-B radiation levels. Rather than addressing a particular species, our model examined the patterns common in most species.

**Table 1:** Summary of the model parameters estimators.

Parameter	Definition	Unit	Range	Assigned values^1^
Leaf optical properties				
1 *k*_*R*_	Total solar UV-B radiation incident on the leaf reflected multiplier	%	0.05–0.7	0.05
2 *k*_*T*_	Total solar UV-B radiation incident on the leaf transmitted multiplier	%	0.01–0.1	0.05
3 *k*_*A, SM*_	UV-B radiation absorbed by pigments multiplier	%	0.94	0.94
4 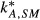	Supplemental increased UV-B radiation absorbed by pigments multiplier	% kJ m^−2^ d^−1^	−0.2 to 1	0.94
CPD/6-4PP induction				
5 *k*_*A, DNA, CPD*_	UV-B radiation reaching the DNA - CPD frequency conversion factor	CPD Mb^−1^ kJ^−1^ m^2^ h	5–74	15
6 *k*_*A, DNA, 6-4PP*_	UV-B radiation reaching the DNA – 6-4PP frequency conversion factor	6-4PP Mb^−1^ kJ^−1^ m^2^ h	0.11–0.67 *CPD*	
7 *k*_*c*_	Correction factor multiplier due to differences in epidermal absorption spectra	%	0.3–1.7	0.65–1.35
CPD photorepair^2^				
8 *a*	CPD photorepair rate multiplier	CPD Mb^−1^ h^−1^	0.3–0.7	
9 *r*_*max*_	Maximum rate of CPD photorepair	CPD Mb^−1^ h^−1^	70–150	
10 *k*_*s*_	Enzyme saturation point	CPD Mb^−1^	300	
11 *k*_*a*_	The level of DNA damage that causes instant cellular apoptosis	CPD Mb^−1^	500	
CPD dark repair^2^				
12 *a*	CPD dark repair rate multiplier	CPD Mb^−1^ h^−1^	0.1–0.3	
13 *r*_*max*_	Maximum rate of CPD dark repair	CPD Mb^−1^ h^−1^	5–7.5	
6-4PP photorepair^2^				
14 *a*	6-4PP photorepair rate multiplier	6-4PP Mb^−1^ h^−1^	0.5–0.9	
15 *r*_*max*_	Maximum rate of 6-4PP photorepair	6-4PP Mb^−1^ h^−1^	70–150	
6-4PP dark repair^2^				
16 *a*	6-4PP dark repair rate multiplier	6-4PP Mb^−1^ h^−1^	0.9	
17 *r*_*max*_	Maximum rate of 6-4PP dark repair	6-4PP Mb^−1^ h^−1^	8–14	
Temperature dependence of CPD induction|6-4PP induction| CPD repair|6-4PP repair mechanisms				
18 *b*_*°*C,0_	Regression equation coefficient	%	0.58|0.57|0.12|0.12	
19 *b*_*°*C,1_	Regression equation coefficient	%°C^*−*1^	0.023|0.021|0.066|0.044	
20 *b*_*°*C,2_	Regression equation coefficient	%°C^*−*2^	−0.0004|−0.0002|−0.0014|−0.0006	
Leaf growth (fast/medium/slow)^3^				
21 *t*_*b,g*_	Time of the beginning of growth	h	Anytime in growing season	
22 *t*_*m,g*_	Time of inflection	h	(*t*_*e,g*_ − *t*_*b,g*_)/2	
23 *t*_*e,g*_	Time of cessation of growth	h	*t*_*b,g*_ + 168|360|720	
Percent of apoptotic cells dependence on the quantity of DNA lesions				
24 *b*_*a,1*_	Regression equation coefficient	% CPD^−1^	0.13	
25 *b*_*a,2*_	Regression equation coefficient	% 6-4PP^−1^	1.09	
26 *k*_*cdd*_	CPD/6-4PP levels over which cell division is delayed	CPD/6-4PP	6–12	5–14
27 *k*_*cdd*_	Duration of the cell division delay	h	8–16	16

1 Where appropriate.

2 *k*_s_ and *k*_a_ have identical values for all repair processes.

3 leaf senescence coefficients were chosen to model identical trends as leaf growth processes and timed for the ending of the growing season considered.

The parameter values resulting in the best fit for the models are presented in Table1. Supplemental model calibration, optimization, and testing can be readily performed as more comprehensive experimental data become available.

## Results

In addition to model analysis simulations, the following scenarios were considered: increased UV-B radiation in combination with different epidermal absorption spectra and CPD repair rates; increased UV-B radiation dose concentrated spread over the leaf expansion period or concentrated in 1, 2, or 3 days; leaves growing in different periods of the growing season under increased UV-B radiation; leaves growing under three temperature regimes under increased UV-B radiation; and slow-, medium-, and fast-growing leaves under increased UV-B radiation regime.

**Figure 2:**
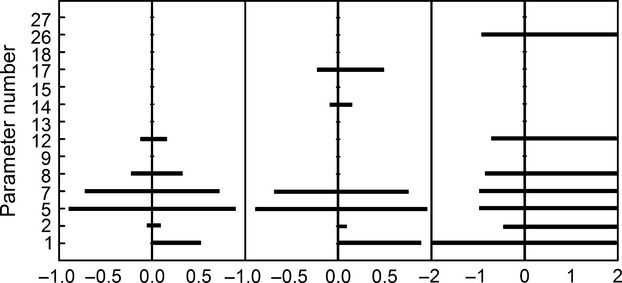
Sensitivity analysis: The relative maximum number of CPDs and 6-4PPs during the leaf growing period and the relative mature leaf area were measured across the leaf optical properties (*k*_*R*_, and *k*_*T*_), CPD induction (*k*_*A, DNA, CPD*_ and *k*_*c*_), CPD/6-4PP photorepair and dark repair (*a* and *r*_*max*_), CPD/6-4PP levels over which cell division is delayed (*k*_*cdd*_), and duration of the cell division delay (*t*_*cdd*_).

The sensitivity analysis showed that the number of 6-4PP induced by UV-B radiation (at either ambient or increased levels) is never high enough to interfere with the leaf growth and development. Also, photorepair of the DNA lesions is never saturated and the differences between the UV-B resistant and sensitive species seem to be in the rate of repair. Increased UV-B radiation does not induce sustained levels of DNA lesions to actually trigger apoptosis in leaf cells. The model was very sensitive to the number of CPD that actually induces cell division delays. In our model, we simulated a range that satisfied the calibration and validation requirements. DNA lesions induction rate was the most influential factor, and it was responsible for the highest variation in CPDs and 6-4PPs numbers, and relative leaf area. Repair of CPDs was less influential on the model, while the model was resistant to changes in 6-4PPs concentrations (Fig.2).

**Figure 3:**
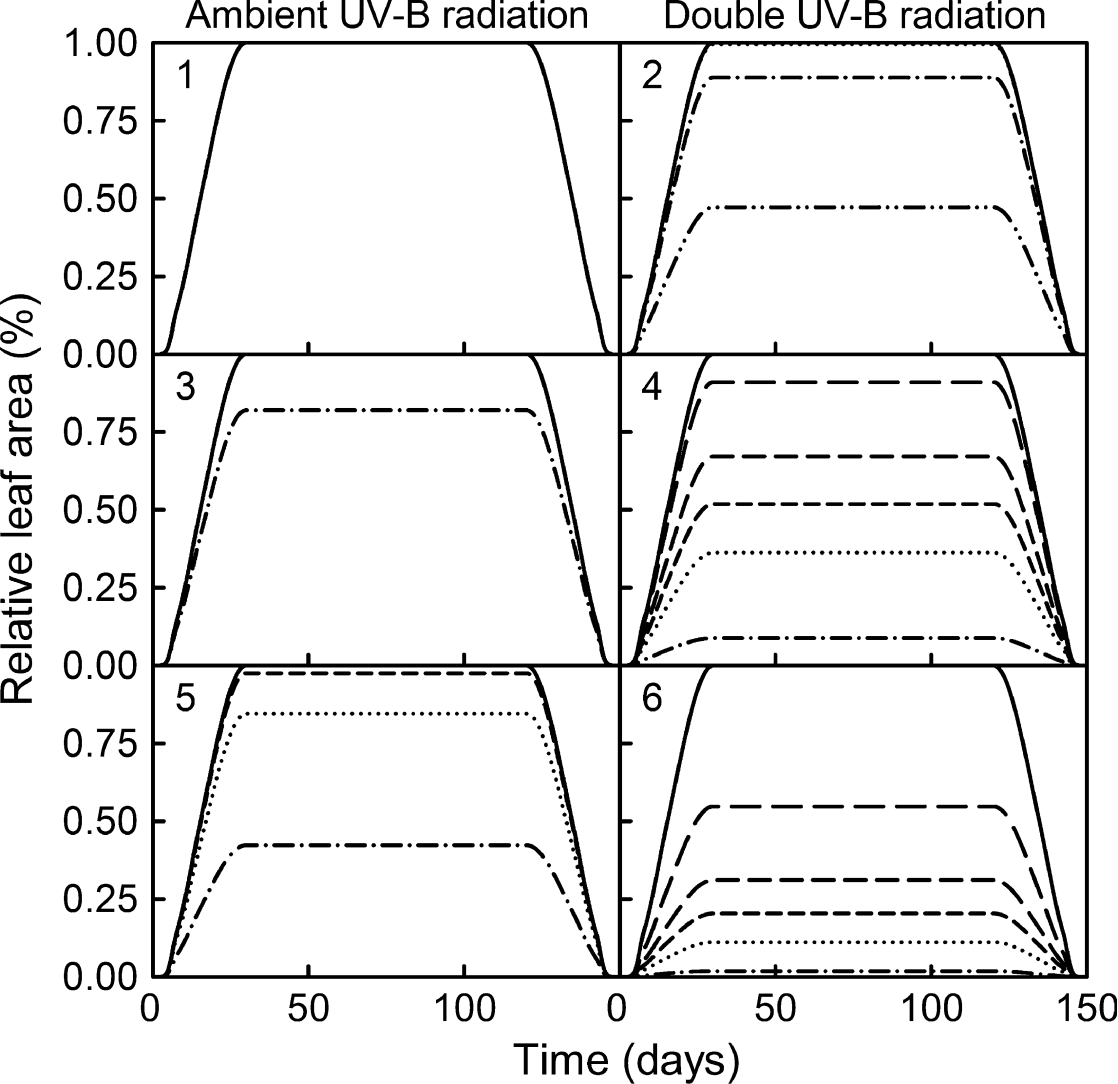
Effect of increased UV-B radiation (ambient and double the UV-B radiation), in combination with relative epidermal absorptance (relative high absorptance at low UV-B radiation wavelengths) (1 and 2), equal absorptance at all UV-B radiation wavelengths (3 and 4), relative high absorptance at low UV-B radiation wavelengths (5 and 6), and CPD repair rates combinations (no CPD inhibition of leaf growth (solid line), high photorepair and high dark repair rates (long dash), high photorepair rate – low dark repair rate (medium dash), average photorepair and dark repair rates (short dash), low photorepair rate – high dark repair rate (dotted line), and low photorepair and dark repair rates (dash-dotted line) on relative leaf area).

Combinations of UV-B radiation doses, epidermal absorptance spectra, and CPD repair rates simulations indicate that plants with relative high epidermal absorptance at short UV-B radiation wavelengths were mostly unaffected by UV-B radiation increases (Fig.3). Only plants with deficient photorepair rates exhibited relative leaf area losses at increased UV-B radiation. Plants with relatively high epidermal absorptance at long UV-B radiation wavelengths were the most sensible to increases in UV-B radiation (Fig.3), while plants with equal epidermal absorptance across wavelengths exhibited intermediary patterns (Fig.3).

**Figure 4:**
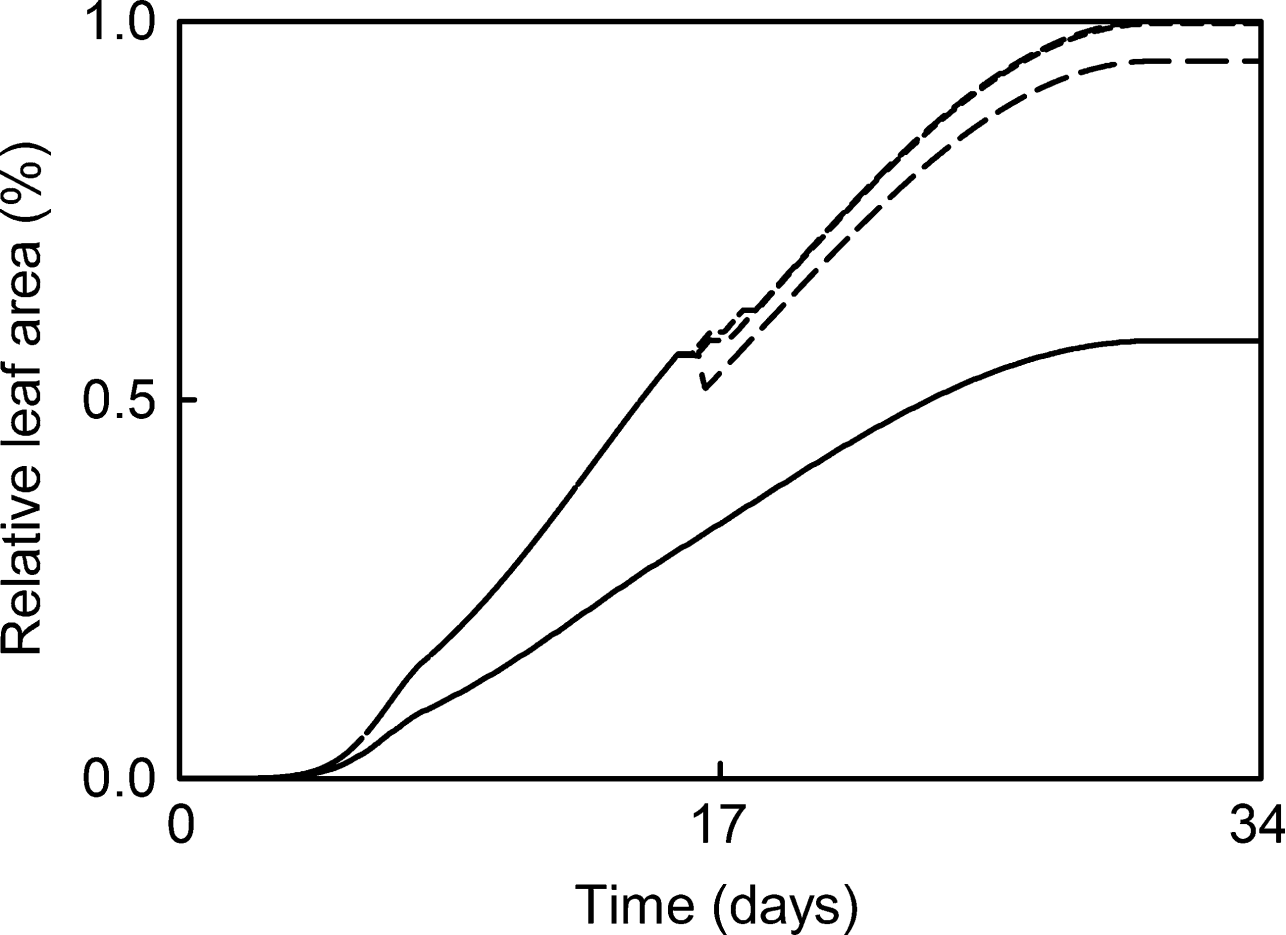
The effect of 100% increased UV-B radiation: spread along the leaf growth period (solid line), in 1-day dose (long dash), in 2 days dose (medium dash), and in 3 days dose (short dash). The simulations were performed for plants with average rates of CPD repairs and equal epidermal absorptance across UV-B radiation wavelengths. Note that the data for 1, 2, and 3 days UV-B radiation doses plots are overlapping until the application of the treatment (in day 18) and became a solid line on the graph.

**Figure 5:**
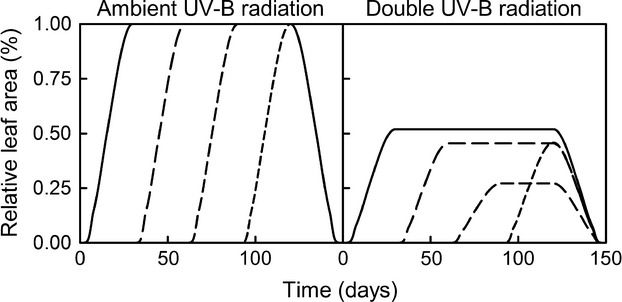
The effect of timing on leaf growth: relative leaf area for leaves growing in May (solid line), June (long dash), July (medium dash), and August dose (short dash), under ambient UV-B and double UV-B. The simulations were performed for plants with average rates of CPD repairs and equal epidermal absorptance across UV-B radiation wavelengths.

When we compared the effect of sustained UV-B radiation increases with short-term increased UV-B bursts, we found that sustained increased UV-B radiation had a higher effect on the final leaf area (Fig.4). The 1-day single dose was the only one that induced a large enough number of CPDs to trigger apoptosis. Also, leaves growing in mid-summer were more affected by increased UV-B radiation than leaves growing in the beginning of the growing season (Fig.5).

**Figure 6:**
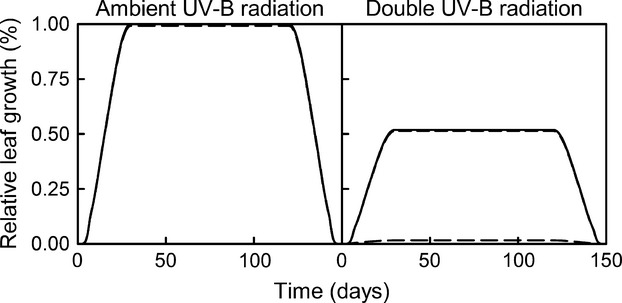
The effect of temperature leaf growth: relative leaf area for leaves under ambient temperatures (solid line), low temperatures: ambient temperatures −10°C (long dash), and high temperatures: ambient temperatures +10°C (medium dash), under ambient UV-B and double UV-B. The simulations were performed for plants growing in May, with average rates of CPD repairs and equal epidermal absorptance across UV-B radiation wavelengths.

**Figure 7:**
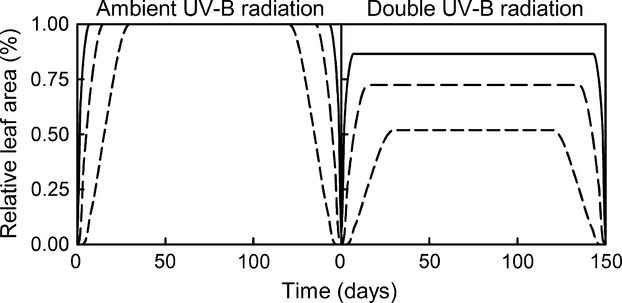
The effect of the duration of leaf growth: relative leaf area for fast-growing leaves: 7 days (solid line), medium growing leaves: 15 days (long dash), and slow-growing leaves: 30 days (medium dash), under ambient UV-B and double UV-B. The simulations were performed for plants growing in May, with average rates of CPD repairs and equal epidermal absorptance across UV-B radiation wavelengths.

Low temperature had an effect on leaf growth, especially when plants were exposed to increased UV-B radiation. Leaves grown at ambient and high temperatures reached similar relative leaf areas (Fig.6). Also, slow-growing leaves exhibited the lowest relative leaf area when exposed to increased UV-B radiation (Fig.7).

## Discussion

Our model simulations showed that UV-B radiation does not induce enough 6-4PPs to interfere directly with the leaf growth and development. This is due to a lower 6-4PP induction rate (Sancar 2003) than for CPD, but also higher photorepair and dark repair (Sancar 2003; Lo et al. 2005; De Lima-Bessa et al. 2008). As 6-4PPs are more readily to trigger apoptosis than CPDs (Lo et al. 2005), a significant finding was that that 6-4PPs might not interfere directly with the leaf growth and development, although they may influence mutagenesis and premature cellular aging (Britt 1996).

The amount of CPD in DNA appeared to be a significant factor for the leaf growth. The number of CPDs is controlled by the quality and quantity of UV-B radiation reaching the DNA (thus, by the absorptance properties on the epidermal secondary metabolites) and by the CPD photorepair and dark repair rates. Regardless of these rates, the model showed that the repair processes do not reach saturation and, given enough time, could repair any amount of damage. While the sustained increased UV-B radiation may be successfully mediated, depending on the epidermal absorptance properties and the rates of repair, occasional extremely high UV-B radiation bursts can be mediated successfully by the repair mechanisms, regardless of their rates.

Moreover, the model showed that increased UV-B radiation did not result in immediate apoptosis of the leaf cells. This simulation does not imply that increased UV-B radiation is instantly lethal to the leaf, but that DNA repair processes were well equipped to handle a severe radiation stress. If the leaf cells are instantaneously apoptotic when exposed to severely high UV-B radiation doses, the mechanisms that induce their death do not seem to be related directly to amount of DNA lesions induced.

Model simulations of increased UV-B radiation in combination with different combinations of the qualitative epidermal absorptance and repair rates (Fig.3) explained why many tree species show little or no significant decreases in leaf size when grown with increased UV-B radiation (Fig. 3.2). Only when coupled with low rates of photorepair, the effects of increased UV-B radiation were highly significant. However, species that exhibit relatively higher absorptance at the long UV-B radiation wavelengths (e.g., most grasses) seem to be more susceptible to UV-B radiation-induced leaf reduction (Figs3, 5 and 7). This confirms previous experimental results that show monocots exhibiting higher sensitivity to increased UV-B radiation than dicots (Barnes et al. 1990). As we simulated equal quantitative epidermal absorptance for all scenarios (Fig.3), the modeled results suggested different plant strategies in dealing with increased UV-B radiation. For example, trees, with leaves present over the entire growing season, seem to have developed UV-B radiation resistance by qualitative changes in epidermal absorptance (i.e., reducing the effective UV-B radiation reaching the DNA). However, grasses seem more susceptible to increased UV-B radiation. Therefore, grass species may cope with increased UV-B radiation by increasing the epidermal pigments concentration, or by avoidance of elevated UV-B radiation seasons.

When we examined the effect of sustained increased UV-B radiation versus similar single doses (Fig.4), we observed that at least from the DNA damage – repair perspective, sustained increased UV-B radiation doses were more detrimental to the leaf area than single extreme doses. A 30-fold UV-B radiation dose for a single day caused sufficient DNA lesions to induce partial leaf cell apoptosis, though the leaf seems to recover shortly. The same dose spread for 2 and 3 days had little to no effect. Again, the model does not account for other cellular damage that might trigger instantaneous apoptosis.

When we simulated leaves growing at different months of the growing season (Fig.5), we observed that ambient UV-B radiation did not have an effect on the final leaf area. It confirms that the timing of leaf growth is controlled by other mechanisms rather than UV-B radiation. But under elevated UV-B radiation, leaves growing in the beginning of the growing season have the least damage.

Simulations on the effect of temperature (Fig.6) showed that the plants were highly vulnerable to the combination of low temperature and increased UV-B radiation. Our model results agree with previous results (Li et al. 1993; Takeuchi et al. 1996; Waterworth et al. 2002). However, it is possible that supplemental epidermal pigments induced by low-temperature environment (Bilger et al. 2007) can successfully complement the diminished repair capacity of cold climate plants.

The duration of the leaf growth appeared to be a factor in the final leaf size (Fig.7). Increased UV-B radiation has to have the least effect on fast-growing plants and the highest effect on slow-growing plants. Therefore, our model predicts that the total UV-B radiation dose during the growing time is the most important factor in the final leaf area.

Improved model predictability can be achieved if some of the model parameters would be estimated for specific species. We recognize that some of the parameters were estimated from maybe dated research, research considering some unrealistic conditions, and research performed on a limited number of plant species, or many times not duplicated. Acknowledging that these estimates might hinder the predictive power of the model, they were considered acceptable (for the purpose of the model), at least until better alternatives are available. Also, the noninclusion of the enhanced UV-B radiation photomorphogenic effects on plant growth and development may have affected the predictive power of the model. Improvements in the model can only be considered when the quantitative relationship between UV-B radiation dose and photomorphogenic responses is better understood. The inclusion of such responses in the model, together with species-specific quantification of UV-B radiation-dependent epidermal absorptance, will allow us to separate and rank the relative importance of those mechanisms in the plant responses to increased UV-B radiation. While we reserve the right to revisit the model in the future, we believe that it is essential to present the model at this stage, despite its shortcomings. First, while the magnitude of effects of UV-B radiation proposed by the results of the model might not be precise, we believe that the direction of the effects and their causes are essentially correct. Second, the model shows the strengths and weaknesses of our understanding of the effects of UV-B radiation in plants.

To name few of those: first, although the epidermal UV-B radiation absorptance is a very important factor in the dynamics of the model, the range of values inferred from the literature was too wide and extrapolation of rates from one range of UV-B doses to a different one is problematic. Second, the conversion factor of the UV-B radiation reaching the DNA in the number of CPDs induced in DNA was estimated through the model calibration processes, from a wide range produced by the literature. Third, while the literature presents a wide array of studies of the photorepair and dark CPD repair rates, most of those studies refer only to rice species and did not offer enough information to detail the Michaelis-Menten enzyme-driven repair models parameters. These parameters are critical in estimating the dynamics of induction and repair of DNA photoproducts and determinant to the associated cell division and leaf expansion processes. Finally, the quantification of the relationship between UV-B radiation dose and photomorphogenic responses is essential for a complete and predictive model.

## Conclusions

This is the first (but probably not the last) mathematical model to integrate the effects of increased UV-B radiation through leaf epidermis, DNA, and leaf growth and development. We intend to revisit the model as more data becomes available. Enhanced UV-B radiation-induced DNA damage significantly delayed cell division until the injury is repaired, resulting in significant reductions in leaf growth and development. A review of the relevant literature showed a wide range of values for the key parameters. Moreover, certain parameter values were inferred only from the calibration process. However, our model allowed the testing of a variety of questions that were difficult to approach through experimental research. Moreover, the model predicts that the total UV-B radiation dose reaching the DNA during the growing time may be an important factor in the final leaf area.

## Supporting Information

**Data S1.** Mathematical model and parameters estimation.

## References

[b1] Aber JD, Bernhardt ES, Dijkstra FA, Gardner RH, Macneale KH, Parton WJ, Canham CD, Cole JJ, Lauenroth WK (2003). Standards of practice for review and publication of models: summary of discussion. Models in ecosystem science.

[b3] Ballare CL, Caldwell MM, Flint SD, Robinson SA, Bornman JF (2011). Effects of solar ultraviolet radiation on terrestrial ecosystems. Patterns, mechanisms, and interactions with climate change. Photochem. Photobiol. Sci.

[b4] Barnes PW, Flint SD, Caldwell MM (1990). Morphological responses of crop and weed species of different growth forms to ultraviolet-B radiation. Am. J. Bot.

[b5] Beemster GT, De Veylder L, Vercruysse S (2005). Genome-wide analysis of gene expression profiles associated with cell cycle transitions in growing organs of *Arabidopsis*. Plant Physiol.

[b6] Bieza K, Lois R (2001). An *arabidopsis* mutant tolerant to lethal ultraviolet-B levels shows constitutively elevated accumulation of flavonoids and other phenolics. Plant Physiol.

[b7] Bilger W, Rolland M, Nybakken L (2007). UV screening in higher plants induced by low temperature in the absence of UV-B radiation. Photochem. Photobiol. Sci.

[b11] Bornman JF, Reuber S, Cen Y-O, Lumsden PJ, Weissenbock G (1997). Ultraviolet radiation as a stress factor and the role of protective pigments. Plants and UV-B: responses to environmental change.

[b12] Britt AB (1996). DNA damage and repair in plants. Annu. Rev. Plant Biol.

[b14] Brown BA, Cloix C, Jiang GH, Kaiserli E, Herzyk P, Kliebenstein DJ (2005). A UV-B-specific signaling component orchestrates plant UV protection. Proc. Natl Acad. Sci. USA.

[b17] Caldwell MM, Bjorn LO, Bornman CH, Flint SD, Kulandaivelu G, Teramura AH (1998). Effects of increased solar ultraviolet radiation on terrestrial ecosystems. J. Photochem. Photobiol., B.

[b20] Chalker-Scott L (1999). Environmental significance of anthocyanins in plant stress responses. Photochem. Photobiol.

[b21] Chen JJ, Mitchell DL, Britt AB (1994). Light-dependent pathway for the elimination of UV-induced pyrimidine-(6-4) pyrimidinone photoproducts in *Arabidopsis*. Plant Cell.

[b23] Cockell CS (2000). The ultraviolet history of the terrestrial planets - implications for biological evolution. Planet. Space Sci.

[b24] Cockell CS, Horneck G (2001). The history of the UV radiation climate of the Earth - theoretical and space-based observations. Photochem. Photobiol.

[b25] Day TA, Demchik SM (1996). Influence of enhanced UV-B radiation on biomass allocation and pigment concentrations in leaves and reproductive structures of greenhouse-grown *Brassica rapa*. Vegetatio.

[b26] Day TA, Neale PJ (2002). Effects of UV-B radiation on terrestrial and aquatic primary producers. Annu. Rev. Ecol. Syst.

[b27] Day TA, Howells BW, Rice WJ (1994). Ultraviolet absorption and epidermal-transmittance spectra in foliage. Physiol. Plant.

[b28] De La Rosa TM, Julkunen-Tiitto R, Lehto T, Aphalo PJ (2001). Secondary metabolites and nutrient concentrations in silver birch seedlings under five levels of daily UV-B exposure and two relative nutrient addition rates. New Phytol.

[b29] De Lima-Bessa KM, Armelini MG, Chigancas V, Jacysyn JF, Amarante-Mendes GP, Sarasin A (2008). CPDs and 6-4PPs play different roles in UV-induced cell death in normal and NER-deficient human cells. DNA Repair (Amst).

[b30] DeLucia EH, Coleman JS, Dawson TE, Jackson RB (2001). Plant physiological ecology: linking the organism to scales above and below - Ecological Society of America Meeting Snowbird, UT, USA, August 2000. New Phytol.

[b32] Dixon RA, Paiva NL (1995). Stress-induced phenylpropanoid metabolism. Plant Cell.

[b34] Gardner RH, Canham CD, Cole JJ, Lauenroth WK, Urban DL (2003). Model validation and testing: past lessons, present concerns, future prospects. Models in ecosystem science.

[b35] Gausman HW, Rodriguez RR, Escobar DE (1975). Ultraviolet radiation reflectance, transmittance, and absorptance by plant leaf epidermises1. Agron. J.

[b36] González R, Mepsted R, Wellburn AR, Paul ND (1998). Non-photosynthetic mechanisms of growth reduction in pea (*Pisum sativum* L.) exposed to UV-B radiation. Plant, Cell Environ.

[b37] Hartmann DL, Wallace JM, Limpasuvan V, Thompson DWJ, Holton JR (2000). Can ozone depletion and global warming interact to produce rapid climate change?. Proc. Natl Acad. Sci.

[b38] Hectors K, Jacques E, Prinsen E, Guisez Y, Verbelen JP, Jansen MA (2010). UV radiation reduces epidermal cell expansion in leaves of *Arabidopsis thaliana*. J. Exp. Bot.

[b39] Hidema J, Kumagai T, Sutherland BM (2000). UV radiation-sensitive Norin 1 rice contains defective cyclobutane pyrimidine dimer photolyase. Plant Cell.

[b40] Hidema J, Song I-K, Sato T, Kumagai T (2001). Relationship between ultraviolet-B sensitivity and cyclobutane pyrimidine dimer photorepair in rice. J. Radiat. Res.

[b41] Hidema J, Taguchi T, Ono T, Teranishi M, Yamamoto K, Kumagai T (2007). Increase in CPD photolyase activity functions effectively to prevent growth inhibition caused by UVB radiation. Plant J.

[b42] Hofmann RW, Campbell BD, Bloor SJ, Swinny EE, Markham K R, Ryan KG (2003). Responses to UV-B radiation in *Trifolium repens* L. - physiological links to plant productivity and water availability. Plant, Cell Environ.

[b43] Hopkins L, Bond MA, Tobin AK (2002). Ultraviolet-B radiation reduces the rates of cell division and elongation in the primary leaf of wheat (*Triticum aestivum* L. cv Maris Huntsman). Plant, Cell Environ.

[b44] Iwamatsu Y, Aoki C, Takahashi M (2008). UVB sensitivity and cyclobutane pyrimidine dimer (CPD) photolyase genotypes in cultivated and wild rice species. Photochem. Photobiol. Sci.

[b45] Jiang C-Z, Yee J, Mitchell DL, Britt AB (1997). Photorepair mutants of *Arabidopsis*. Proc. Natl Acad. Sci.

[b46] Kang HS, Hidema J, Kumagai T (1998). Effects of light environment during culture on UV-induced cyclobutyl pyrimidine dimers and their photorepair in rice (*Oryza sativa* L.). Photochem. Photobiol.

[b48] Koes RE, Quattrocchio F, Mol JNM (1994). The flavonoid biosynthetic-pathway in plants - function and evolution. BioEssays.

[b49] Kolb CA, Kaser MA, Kopecky J, Zotz G, Riederer M, Pfundel EE (2001). Effects of natural intensities of visible and ultraviolet radiation on epidermal ultraviolet screening and photosynthesis in grape leaves. Plant Physiol.

[b50] Lavola ANU, Julkunen-Tiitto R, Aphalo P, De La Rosa T, Lehto T (1997). The effect of UV-B radiation on UV-absorbing secondary metabolites in birch seedlings grown under simulated forest soil conditions. New Phytol.

[b51] van der Leun J, Tevini M, Tang X, Worrest RCE (1998). Environmental effects of ozone depletion: 1998 update.

[b52] Li J, Ou-Lee TM, Raba R, Amundson RG, Last RL (1993). *Arabidopsis* flavonoid mutants are hypersensitive to UV-B irradiation. Plant Cell.

[b53] Li SS, Paulsson M, Bjorn LO (2002). Temperature-dependent formation and photorepair of DNA damage induced by UV-B radiation in suspension-cultured tobacco cells. J. Photochem. Photobiol., B.

[b54] Liu L, Gitz DC, Mcclure JW (1995). Effects of UV-B on flavonoids, ferulic acid, growth and photosynthesis in barley primary leaves. Physiol. Plant.

[b55] Lo HL, Nakajima S, Ma L, Walter B, Yasui A, Ethell DW (2005). Differential biologic effects of CPD and 6-4PP UV-induced DNA damage on the induction of apoptosis and cell-cycle arrest. BMC Cancer.

[b56] Lowry B, Lee D, Hebant C (1980). The origins of land plants: a new look at an old problem. Taxon.

[b58] Meijkamp B, Aerts R, Van Der Staaij J, Tosserams M, Ernst W, Rozema J, Rozema J (1999). Effects of UV-B on secondary metabolites on plants. Stratospheric ozone depletion: the effects of enhanced UV-B radiation on terrestrial ecosystems.

[b59] Milchunas DG, King JY, Mosier AR, Moore JC, Morgan JA, Quirk MH (2004). UV radiation effects on plant growth and forage quality in a shortgrass steppe Ecosystem. Photochem. Photobiol.

[b63] Olsson LC, Veit M, Weissenbock G, Bornman JF (1998). Differential flavonoid response to enhanced UV-B radiation in *Brassica napus*. Phytochemistry.

[b65] Plentinger MC, Penning De Vries FWT (1996).

[b66] Qi Y, Bai S, Heisler GM (2003). Changes in ultraviolet-B and visible optical properties and absorbing pigment concentrations in pecan leaves during a growing season. Agric. For. Meteorol.

[b67] Quaite FE, Takayanagi S, Ruffini J, Sutherland JC, Sutherland BM (1994). DNA damage levels determine cyclobutyl pyrimidine dimer repair mechanisms in alfalfa seedlings. Plant Cell.

[b68] Rettberg P, Horneck G, Strauch W, Facius R, Seckmeyer G (1998). Simulation of planetary UV radiation climate on the example of the early Earth. Adv. Space Res.

[b69] Robberecht R, Caldwell MM (1978). Leaf epidermal transmittance of ultraviolet-radiation and its implications for plant sensitivity to ultraviolet-radiation induced injury. Oecologia.

[b70] Robberecht R, Caldwell MM, Billings WD (1980). Leaf ultraviolet optical properties along a latitudinal gradient in the arctic-alpine life zone. Ecology.

[b71] Rousseaux MC, Flint SD, Searles PS, Caldwell MM (2004). Plant responses to current solar ultraviolet-B radiation and to supplemented solar ultraviolet-B radiation simulating ozone depletion: an experimental comparison. Photochem. Photobiol.

[b72] Rozema J, Van de Staaij JWM, Lumsden PJ, Tosserams M (1997). Effects of UV-B radiation on plants from agro- and natural ecosystems. Plants and UV-B: responses to environmental change.

[b73] Rozema J, Van De Staaij J, Bjorn LO, Rozema J, De Bakker N (1999). Depletion of stratospheric ozone and solar UV-B radiation: evolution of land plants, UV-screens and function of polyphenolics. Stratospheric ozone depletion: the effects of enhanced UV-B radiation on terrestrial ecosystems.

[b74] Ryan KG, Markham KR, Bloor SJ, Bradley JM, Mitchell KA, Jordan BR (1998). UVB radiation induced increase in quercetin: kaempferol ratio in wild-type and transgenic lines of Petunia. Photochem. Photobiol.

[b75] Ryan KG, Swinny EE, Markham KR, Winefield C (2002). Flavonoid gene expression and UV photoprotection in transgenic and mutant Petunia leaves. Phytochemistry.

[b76] Rykiel JEJ (1996). Testing ecological models: the meaning of validation. Ecol. Model.

[b77] Sagan C (1973). Ultraviolet selection pressure on earliest organisms. J. Theor. Biol.

[b78] Sancar A (2003). Structure and function of DNA photolyase and cryptochrome blue-light photoreceptors. Chem. Rev.

[b79] Sancar A, Sancar GB (1988). DNA-repair enzymes. Annu. Rev. Biochem.

[b80] Schmelzer E, Jahnen W, Hahlbrock K (1988). In situ localization of light-induced chalcone synthase mRNA, chalcone synthase, and flavonoid end products in epidermal cells of parsley leaves. Proc. Natl Acad. Sci.

[b81] Searles PS, Flint SD, Caldwell MM (2001). A meta analysis of plant field studies simulating stratospheric ozone depletion. Oecologia.

[b82] Sheahan JJ (1996). Sinapate esters provide greater UV-B attenuation than flavonoids in *Arabidopsis thaliana* (Brassicaceae). Am. J. Bot.

[b83] Shugart HH (1984). A theory on forest dynamics. The ecological implications of forest succesion models.

[b84] Sisson WB (1981). Photosynthesis, growth, and ultraviolet irradiance absorbance of *Cucurbita pepo* L. leaves exposed to ultraviolet-B radiation (280–315 nm). Plant Physiol.

[b85] Srivastava LM (2002). Plant growth and development: hormones and environment.

[b87] Stafford HA (1991). Flavonoid evolution - an enzymatic approach. Plant Physiol.

[b88] Systems V (2009). http://www.vensim.com.

[b89] Takeuchi Y, Murakami M, Nakajima S, Kondo S, Nikaido O (1996). Induction and repair of damage to DNA in cucumber cotyledons irradiated with UV-B. Plant Cell Physiol.

[b90] Taylor RM, Tobin AK, Lumsden PJ, Bray CM (1997). DNA damage and repair in plants. Plants and UV-B responses to environmental change.

[b91] Team RDC (2010). R: A language and environment for statistical computing.

[b92] Tegelberg R, Veteli T, Aphalo PJ, Julkunen-Tiitto N (2003). Clonal differences in growth and phenolics of willows exposed to elevated ultraviolet-B radiation. Basic Appl. Ecol.

[b93] Teramura AH, Ziska LH, Sztein AE (1991). Changes in growth and photosynthetic capacity of rice with increased UV-B radiation. Physiol. Plant.

[b94] Tevini M, Iwanzik W, Thoma U (1981). Some effects of enhanced UV-B irradiation on the growth and composition of plants. Planta.

[b95] Tevini M, Thoma U, Bauer H, Caldwell MM, Tevini M, Worrest RC, Iwanzik W (1982). Effect of enhanced UV-B radiation on development and composition of plants. Biological effects of UV-B radiation: workshop: papers.

[b96] Tevini M, Thoma U, Iwanzik W (1983). Effects of enhanced UV-B radiation on germination, seedling growth, leaf anatomy and pigments of some crop plants. Z. Pflanzenphysiol.

[b97] United Nations Environment Programme EEAP (2012). Environmental effects of ozone depletion and its interactions with climate change: progress report, 2011. Photochem. Photobiol. Sci.

[b98] Vandestaaij JWM, Ernst WHO, Hakvoort HWJ, Rozema J (1995). Ultraviolet-B (280–320 Nm) absorbing pigments in the leaves of silene vulgaris - their role in UV-B tolerance. J. Plant Physiol.

[b99] Wargent JJ, Gegas VC, Jenkins GI, Doonan JH, Paul ND (2009a). UVR8 in *Arabidopsis thaliana* regulates multiple aspects of cellular differentiation during leaf development in response to ultraviolet B radiation. New Phytol.

[b100] Wargent JJ, Moore JP, Roland Ennos A, Paul ND (2009b). Ultraviolet radiation as a limiting factor in leaf expansion and development. Photochem. Photobiol.

[b101] Warren JM, Bassman JH, Eigenbrode S (2002). Leaf chemical changes induced in *Populus trichocarpa* by enhanced UV-B radiation and concomitant effects on herbivory by *Chrysomela scripta* (Coleoptera: Chrysomidae). Tree Physiol.

[b102] Waterworth WM, Jiang O, West CE, Nikaido M, Bray C M (2002). Characterization of *Arabidopsis* photolyase enzymes and analysis of their role in protection from ultraviolet-B radiation. J. Exp. Bot.

[b103] Webb AR, Lumsden PJ (1997). Monitoring changes in UV-B radiation. Plants and UV-B: responses to environmental change.

[b104] Weber S (2005). Light-driven enzymatic catalysis of DNA repair: a review of recent biophysical studies on photolyase. Biochimica Et Biophysica Acta-Bioenergetics.

[b105] Winkel-Shirley B (2002). Biosynthesis of flavonoids and effects of stress. Curr. Opin. Plant Biol.

