# Supplementary material for: Integration and scaling of UV-B radiation effects on plants: from DNA to leaf
Source: Ecol Evol. 2015 Jun 2;5(13):2544–55. doi: 10.1002/ece3.1332 (PMC4523352; doi:10.1002/ece3.1332)
Supplement: Supplementary file 1 [file ece30005-2544-sd1.docx]

**SUPPORTING INFORMATION:**

**MATHEMATICAL MODEL AND PARAMETERS ESTIMATION**

**1. MATHEMATICAL MODEL**

The dynamics of the UV-B radiation pathway in the leaf, the consequences for cell processes, and leaf morphology were expressed mathematically as follows.

**1.1 UV-B radiation**

Ultraviolet-B radiation data were obtained from the UV-B Monitoring and Research Program (UVMRP) over the period 2000-2009, for nearest location, Pullman, Washington. We used UV-B Langley calibrated data, considered more appropriate than lamp calibrated data for sunny and dry locations ([USDA, 2010](#_ENREF_52)). Ultraviolet-B radiation data were averaged for the 10-year period, and for each month of the local growing season (May-September). Averaged hourly temperature data were obtained for Spokane, Washington from National Oceanic and Atmospheric Administration - National Climatic Data Center ([NOAA, 2011](#_ENREF_31)).

**1.2 Leaf optical properties**

UV-B radiation reaching a leaf reflected, absorbed, or transmitted.

$E=E_{R}+E_{A}+E_{T}$ (S1)

Where, $E$ is the total solar UV-B radiation incident to the leaf, $E_{R}$ total solar UV-B radiation reflected by the leaf, $E_{A}$ total solar UV-B radiation absorbed by the leaf, and $E_{T}$ total solar UV-B radiation transmitted through the leaf.

Fractions of the total solar UV-B radiation incident on the leaf are reflected and transmitted:

$E_{R}=k_{R}E$ (S2)

$E_{T}=k_{T}E$ (S3)

Where $k_{R}$ and $k_{T}$ are the total solar UV-B radiation incident on the leaf reflected and transmitted multipliers.

Solar UV-B radiation is absorbed by secondary metabolites, DNA and other leaf structures. The current model assumes that the fraction of the UV-B radiation not absorbed by secondary metabolites is entirely absorbed by DNA. Although other leaf structures and cell components are important receptors of UV-B radiation, in the absence of quantitative evaluations of their relative absorptance, we made the assumption that DNA is the major recipient because of its key role in the sensitivity of plant species to UV-B radiation.

$E_{A}=E_{A, SM}+E_{A,DNA}$ (S4)

Where, $E_{A, SM}$ is the UV-B radiation absorbed by secondary metabolites, and $E_{A,DNA}$ is the UV-B radiation absorbed by DNA.

The UV-B radiation absorbed by secondary metabolites was expressed as:

$E_{A,SM}=k_{A,SM}E_{A}$ (S5)

Where $k_{A,SM}$ is the UV-B radiation absorbed by the secondary metabolites multiplier.

The radiation absorbed by secondary metabolites $E_{A, SM}$ is proportional to the quantity of secondary metabolites, and changes accordingly.

**1.3 UV-B radiation induced DNA damage and repair**

The general model for UV-B radiation induced damage in a leaf cell is as follows:

$D_{DNA, CPD/6-4PP}=D_{I,CPD/6-4PP}-D_{PR,CPD/6-4PP}-D_{ER,CPD/6-4PP}$ (S6)

Where $D_{DNA}$ represent the CPD/6-4PPs frequency present in the DNA, $D_{I}$ are the CPD/6-4PPs frequencies induced by the UV-B radiation reaching the DNA, $D_{PR}$ and $D_{ER}$ are the CPD/6-4PPs frequencies photorepaired and excision repaired, respectively (CPD/6-4PPs Mb^-1^).

Since the induced CPD/6-4PPs frequencies are UV-B radiation dose dependent, and spectra dependent, the CPD/6-4PPs frequency induced$D_{I}$ becomes:

$D_{I}=k_{A,DNA}k_{c}E_{A,DNA}$ (S7)

Where, $k_{A,DNA}$ is the UV-B radiation reaching the DNA - CPD/6-4PPs frequency conversion factor, and $k_{c}$ is a correction factor multiplier due to differences in absorption spectra of epidermal secondary metabolites.

To evaluate $k_{c}$ the DNA weighted UV-B radiation relationships ([Caldwell *et al.*, 1983](#_ENREF_3), [Setlow, 1974](#_ENREF_42)), were used for various absorption scenarios ([Day *et al.*, 1994](#_ENREF_6), [Lavola *et al.*, 1997](#_ENREF_23), [Qi *et al.*, 2003](#_ENREF_34), [Schmelzer *et al.*, 1988](#_ENREF_41), [Sisson, 1981](#_ENREF_44)).

The total DNA weighted UV-B exposure is given by

$E_{A,DNA, weighted}=\int_{T_{1}}^{T_{2}} \int_{280}^{320} {S_{\lambda}E}_{A,DNA,\lambda}d\lambda dt$ (S8)

and the action spectra for DNA damage was given by ([Caldwell *et al.*, 1983](#_ENREF_3), [Setlow, 1974](#_ENREF_42)).

$S_{\lambda}=e^{13.82\left( \frac{1}{1+e^{\frac{\lambda-310}{9}}}-1 \right)}$ (S9)

Where, $S_{\lambda}$ is the action spectra for DNA damage ([Caldwell *et al.*, 1983](#_ENREF_3), [Setlow, 1974](#_ENREF_42)), $E_{A,DNA,\lambda}$(Wm^-2^ nm^-1^) is the radiant flux density incident on the surface per unit of wavelength interval reaching the DNA, $\lambda$(nm) is the wavelength, $T_{1}$ and $T_{2}$ is the time interval the total exposure is calculated.

Both the CPD/6-4PPs frequencies photorepaired ($D_{PR}$), and excision repaired ($D_{ER}$) are proportional to the level of damage induced ([Hidema *et al.*, 2001](#_ENREF_11), [Hidema *et al.*, 1997](#_ENREF_14), [Taylor *et al.*, 1997](#_ENREF_47)).

$D_{{PR}/{ER,{CPD}/{6-4PP}}}=rD_{I,CPD/6-4PP}$ (S10)

Since photorepair and excision repair mechanisms are enzyme mediated, the rates of repair were considered to follow a basic Michaelis-Menten model ([Lodish *et al.*, 2008](#_ENREF_28)) until the CPD/6-4 PP photolyase reach a level of saturation, followed to a decline in rates to zero, which is the instant cell apoptosis corresponding to the level of damage that disturbs instantaneous the cell activity (Figure S1).


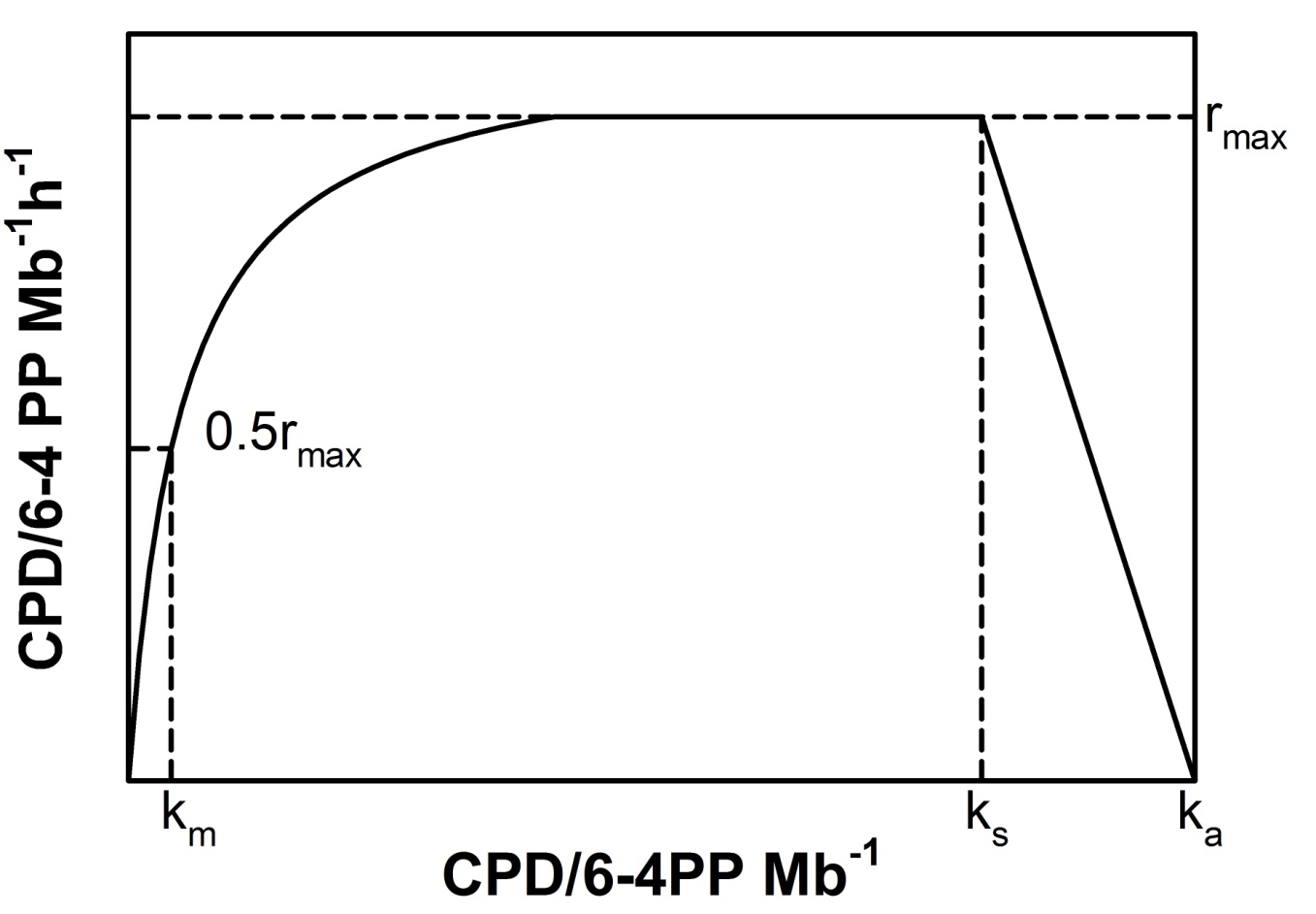


Figure S1: Conceptual model of DNA repair rate as a function of concentration of CPD/6-4PP concentration. Repair rates follow a basic Michaelis-Menten model ([Lodish *et al.*, 2008](#_ENREF_28)) until the photolyase reach a level of saturation, followed to a decline in rates to zero, corresponding to the level of damage that disturbs instantaneous the cell activity. Note: the processes expressed are not at real scale.

This relationship is adjusted accordingly for photorepair: in the absence of PAR radiation the rate of repair is zero.

$r=\left\{ \begin{aligned} \frac{r_{max}D_{I,CPD/6-4PP}}{k_{m}+D_{I,CPD/6-4PP}} {0\leq D}_{I,CPD/6-4PP}\leq k_{s} \\ b_{0}-b_{1}D_{I,CPD/6-4PP} {k_{s}<D}_{I,CPD/6-4PP}\leq k_{a} \end{aligned} \right.$ (S11)

Where, $r_{max}$ is the maximum rate of repair, $k_{m}$ is the Michaelis constant (the concentration of substrate that gives exactly a rate half of $r_{max}$), $k_{s}$ is the enzyme saturation point, $k_{a}$ is the level of DNA damage that causes instant cellular apoptosis , b_0_ and b_1_ are the linear regression parameters for repair rate decline (Figure S1).

The temperature dependence of both CPD/6-4 PP induction and repair were considered to follow a polynomial relationship of the form:

$r\left( \% \right)=b_{℃,0}+b_{℃,1}℃+b_{℃,2}℃^{2}$ (S12)

Where, $r\left( \% \right)$ is the CPD/6-4 PP induction/repair rates, $b_{℃,0}$, $b_{℃,1}$, and $b_{℃,2}$ the coefficients of the polynomial relationship, and $℃$ is the temperature ($℃$).

**1.4 Leaf growth and development**

The processes governing leaf progression were grouped in three major stages: expansion, longevity, and senescence. Leaf expansion refers to the period when leaf increases its surface from the leaf primordium to the maximum area of the leaf. Longevity refers to the period beginning with leaf expansion until complete senescence. Leaf senescence refers to the period when the leaf starts to exhibit chlorophyll loss until cell-leaf death ([Nooden, 2004](#_ENREF_32), [Srivastava, 2002](#_ENREF_45)).

To model the leaf growth, we chose the beta sigmoid function, which has few, unique, and readily interpretable parameters ([Muller *et al.*, 2006](#_ENREF_30), [Yin *et al.*, 2003](#_ENREF_56)). In the beta function the starting and ending times of growth and senescence are clearly defined, and it is a function of seven biologically relevant parameters (Figure S2).


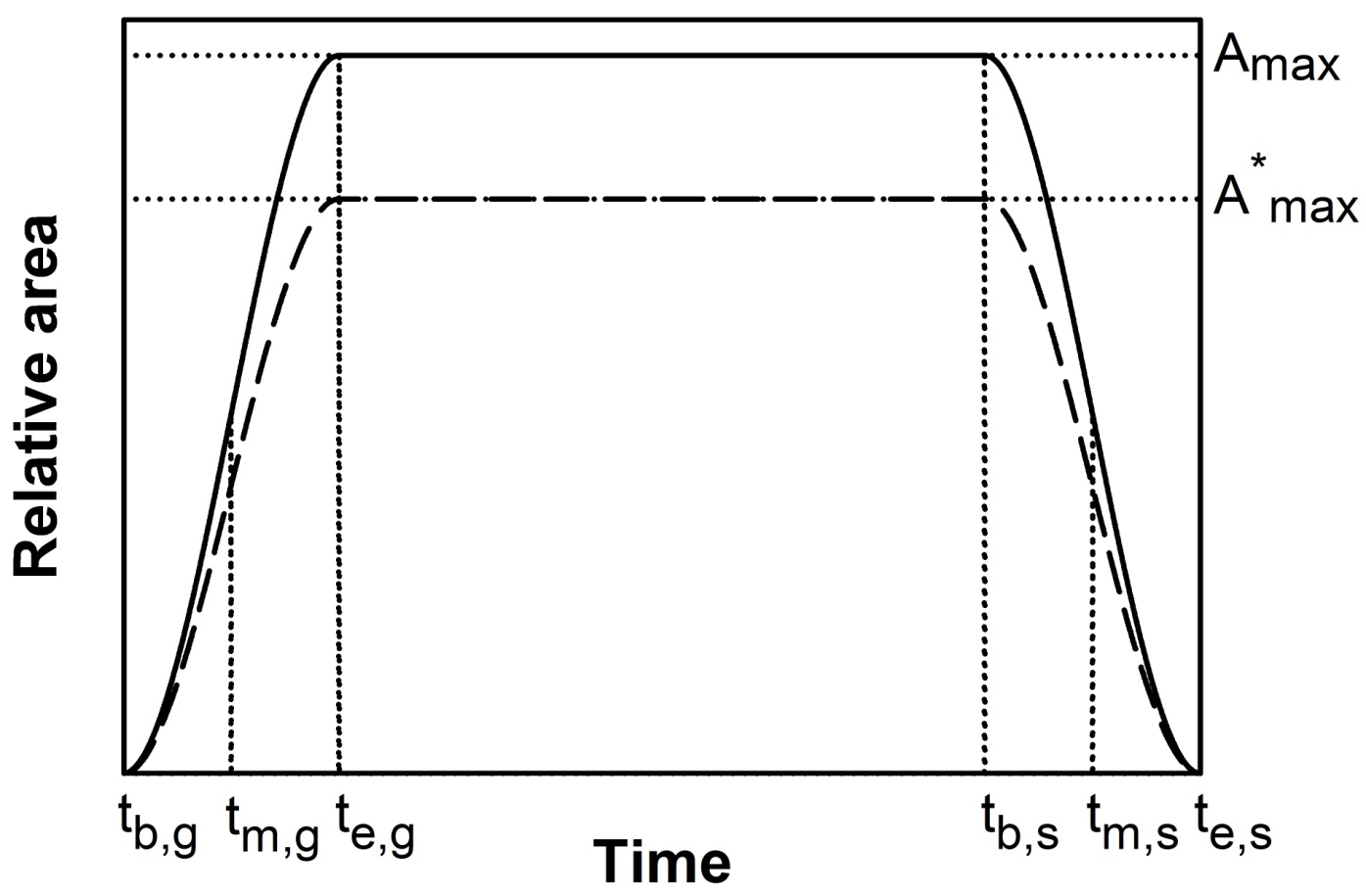


Figure S2: Dynamics of leaf area using the beta sigmoid function: normal leaf – solid line (with maximum area$A_{max})$; hypothetical leaf with UVB-induced DNA damage during growth – dashed line (with maximum area$A_{max}^{*})$. Note: the chlorophyll loss during the senescence period is expressed as effective loss of leaf area ([Muller *et al.*, 2006](#_ENREF_30), [Yin *et al.*, 2003](#_ENREF_56)).

Thus, leaf area dynamics (Figure S2) were simulated as follows:

$A=\left\{ \begin{matrix} \begin{matrix} \begin{matrix} 0 & if t<t_{b,g} \end{matrix} \\ \begin{matrix} A_{max}\left( 1+\frac{t_{e,g}-t}{t_{e,g}-t_{m,g}} \right)\left( \frac{t-t_{b,g}}{t_{e,g}-t_{b,g}} \right)^{\frac{t_{e,g}-t_{b,g}}{t_{e,g}-t_{m,g}}} & if t_{b,g}\leq t\leq t_{e,g} \end{matrix} \end{matrix} \\ \begin{matrix} \begin{matrix} A_{max} & if t_{e,g}\leq t\leq t_{b,s} \end{matrix} \\ \begin{matrix} A_{max}\left[ 1-\left( 1+\frac{t_{e,s}-t}{t_{e,s}-t_{m,s}} \right)\left( \frac{t-t_{b,s}}{t_{e,s}-t_{b,s}} \right)^{\frac{t_{e,s}-t_{b,s}}{t_{e,s}-t_{m,s}}} \right] & if t_{b,s}\leq t\leq t_{e,s} \end{matrix} \\ \begin{matrix} 0 & if t>t_{e,s} \end{matrix} \end{matrix} \end{matrix} \right.$ (S13)

Where, $t_{b,g}$, $t_{m,g}$ and $t_{e,g}$ are time when growth begins, time of inflection, and time of cessation of growth, respectively; $t_{b,s}$, $t_{m,s}$ and $t_{e,s}$ are time when senescence begins, time of inflection, and time of cessation of senescence, respectively; $A_{max}$ is the maximum relative leaf area.

Leaf growth was expressed as a discrete process:

$A_{t+1}=\lambda(t) A_{t}$ (S14)

Where, A_t_ and A_t+1_ are leaf area at time t and t+1, respectively; $\lambda(t)$ is the time-dependent rate of increase, derived from Equation S13. These rates of increase were corrected according to the level of DNA damage.

Leaf growth process was considered to be driven initially by active cell division, followed by a decrease in the number of dividing cells, active cell expansion and differentiation, and leaf maturity ([Beemster *et al.*, 2005](#_ENREF_1)). Thus, increased UV-B radiation was considered to cause delays in cell division and expansion, during the leaf growth process (i.e., reduced $\lambda(t)$, time-dependent rates of leaf increase).

**2. PARAMETER ESTIMATION**

**2.1 UV-B radiation**

The ten year averaged UV-B radiation for the Pullman, Washington station of the UV-B Monitoring and Research Program (UVMRP) was considered the baseline UV-B radiation environment. Increases of 100% in UV-B radiation scenarios were considered in our simulations. The parameter estimates are presented in Table 1.

**2.2 Leaf optical properties**

The range for the leaf reflectance was considered $k_{R}=0.05-0.7$ of the incident solar UV-B radiation, while the one for transmittance was $k_{T}=0.01-0.1$ ([Gausman *et al.*, 1975](#_ENREF_9), [Robberecht & Caldwell, 1978](#_ENREF_36), [Robberecht *et al.*, 1980](#_ENREF_37)).

The epidermal pigments absorption was considered $k_{A,SM}=0.94$ ([Robberecht & Caldwell, 1978](#_ENREF_36)). Changes in epidermal pigments absorption with increased UV-B radiation were considered to range between $k_{A,SM}^{*}=-0.2-1$ per kJ m^-2^ d^-1^ ([Bornman *et al.*, 1997](#_ENREF_2), [Day & Demchik, 1996](#_ENREF_5), [de la Rosa *et al.*, 2001](#_ENREF_7), [Kolb *et al.*, 2001](#_ENREF_21), [Li *et al.*, 1993](#_ENREF_24), [Liu *et al.*, 1995](#_ENREF_26), [Meijkamp *et al.*, 1999](#_ENREF_29), [Olsson *et al.*, 1998](#_ENREF_33), [Sheahan, 1996](#_ENREF_43), [Tegelberg *et al.*, 2003](#_ENREF_48), [Tevini *et al.*, 1981](#_ENREF_49), [Tevini *et al.*, 1982](#_ENREF_50), [Tevini *et al.*, 1983](#_ENREF_51), [Vandestaaij *et al.*, 1995](#_ENREF_53)).

**2.3 UV-B radiation induced DNA damage and repair**

Since the UV-B radiation induced damage to DNA is a photochemical process, the rate of CPD induction should be similar for most species. Studies on rice varieties cultivated under laboratory conditions indicate that a dose of unweighted UV-B radiation of 1 kJ m^-2^ at the leaf surface induces approximately 4 CPDs Mb^-1^ ([Hidema & Kumagai, 1998](#_ENREF_12), [Hidema *et al.*, 2000](#_ENREF_13), [Hidema *et al.*, 1997](#_ENREF_14), [Takeuchi *et al.*, 1996](#_ENREF_46)), depending on the growth conditions and UV-B action spectra. To quantify the rate of CPD induction as a function of the dose of UV-B radiation reaching the DNA, we considered two extreme scenarios regarding the UV-B absorptance of epidermal secondary metabolites. Firstly, an epidermal absorptance of 0.94 leads to a $k_{A,DNA}=74$ CPD Mb^-1^ kJ^-1^ m^2^ h. We consider this value as an overestimation of the true value, since plants in these studies were cultivated without UV-B radiation exposure, and the doses used to induce CPDs were over 10-20 times greater than the ambient conditions. Secondly, if we consider an epidermal absorptance of about 0.03 – resulting from quantifications of secondary metabolites in rice species grown with and without UV-B radiation supplementation, and the expected epidermal absorptance under ambient UV-B radiation conditions ([Hidema *et al.*, 1997](#_ENREF_14), [Kang *et al.*, 1998](#_ENREF_20), [Kon *et al.*, 2004](#_ENREF_22), [Robberecht & Caldwell, 1978](#_ENREF_36)), we come with a value of $k_{A,DNA}=5$ CPD Mb^-1^ kJ^-1^ m^2^ h. The second value we believe to be an underestimation of the true value due to the poor understanding of the dynamics of secondary metabolites in epidermis, at different UV-B radiation exposures. The range considered was $k_{A,DNA}=5-74$ CPD Mb^-1^ kJ^-1^ m^2^ h.

We assumed that low UV-B radiation produces CPD to 6-4PP ratio of 9:1, and high UVB doses produce ratios of 6:4 ([Sancar, 2003](#_ENREF_40)), since no published data were available. This model used the following arbitrary rule: UV-B radiation induced CPD to 6-4PP ratio is 9:1 for the 1^st^ quartile of the overall UV-B radiation for the growing season, 8:2 for the 2^nd^ quartile, 7:3 for the 3^rd^ quartile, and 6:4 for the 4^th^ quartile.

Species with maximum absorption at shorter wavelengths (Figure S3) had up to 70% less DNA weighted UV-B radiation reaching the DNA than the species exhibiting equal absorptance across wavelengths, while species with maximum absorption at longer wavelengths (Figure S3) had up to 70% higher DNA weighted UV-B radiation reaching the DNA than the species exhibiting equal absorptance across wavelengths. These translates for initial values for $k_{c}$ values of 0.3 to 1.7 depending on the absorption trend considered, with $k_{c}=1$ for species with equal epidermal absorptance across all UV-B wavelengths.


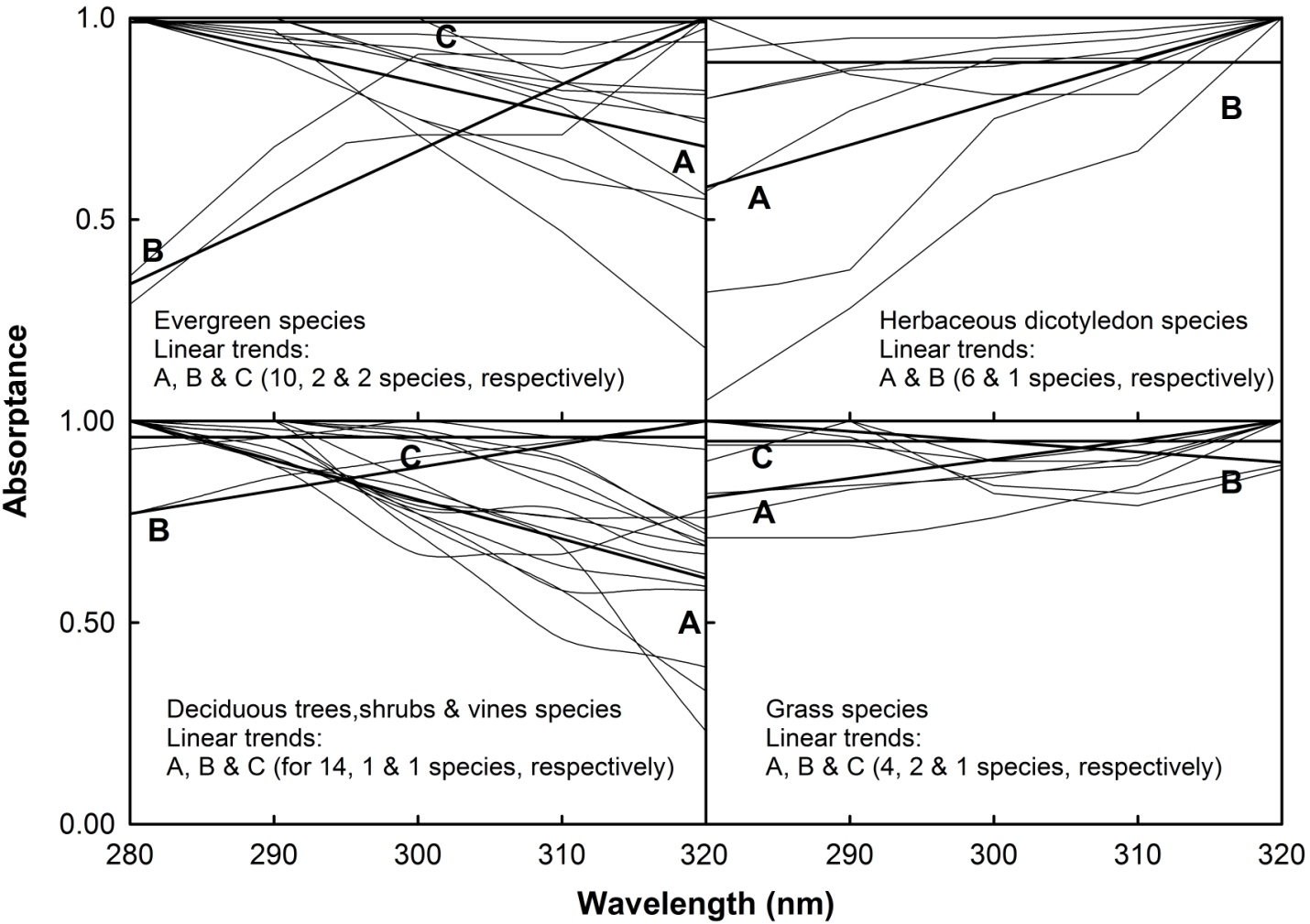


Figure S3: Relative absorption of secondary metabolites for evergreens, deciduous trees, shrubs, vines, herbaceous dicotyledons and grass species. The thin lines indicate the relative absorptance of individual species, while the bold lines (A, B, and C) indicate the general linear trends derived from the relative absorptance for individual species. Inferred from ([Day *et al.*, 1994](#_ENREF_6), [Lavola *et al.*, 1997](#_ENREF_23), [Qi *et al.*, 2003](#_ENREF_34), [Schmelzer *et al.*, 1988](#_ENREF_41), [Sisson, 1981](#_ENREF_44)).

The Michaelis-Menten photorepair model parameters could not be inferred from the studies considered ([Hidema *et al.*, 2001](#_ENREF_11), [Hidema *et al.*, 1997](#_ENREF_14), [Hidema *et al.*, 2007](#_ENREF_15), [Iwamatsu *et al.*, 2008](#_ENREF_18), [Kang *et al.*, 1998](#_ENREF_20), [Quaite *et al.*, 1994](#_ENREF_35)), since in most of these studies the enzyme saturation was not reached. Therefore, the Michaelis-Menten photorepair model was approximated with a linear rate of repair increase as a function of CPD concentration followed by maximum rate of repair (corresponding to enzyme saturation). Thus, equation S11 becomes:

$r=\left\{ \begin{aligned} {aD}_{I,{CPD}/{6-4PP}} {0\leq D}_{I,{CPD}/{6-4PP}}\leq{r_{max}}/a \\ r_{max} {{r_{max}}/a\leq D}_{I,{CPD}/{6-4PP}}\leq k_{s} \\ b_{0}-b_{1}D_{I,{CPD}/{6-4PP}} {k_{s}<D}_{I,{CPD}/{6-4PP}}\leq k_{a} \\ 0 {k_{s}<D}_{I,{CPD}/{6-4PP}} \end{aligned} \right.$ (S15)

The proposed values for each CPD photorepair and dark repair mechanisms are presented in Table 1 ([Hidema *et al.*, 2001](#_ENREF_11), [Hidema *et al.*, 1997](#_ENREF_14), [Hidema *et al.*, 2007](#_ENREF_15), [Iwamatsu *et al.*, 2008](#_ENREF_18), [Kang *et al.*, 1998](#_ENREF_20), [Quaite *et al.*, 1994](#_ENREF_35)). The estimation of $k_{s}$ (enzyme saturation point), and $k_{a}$ (level of DNA damage that causes instant cellular apoptosis) was more difficult. In *Oryza* and *Medicado* varieties, the rate of CPD repair was not inhibited at induced levels of $50-70 CPD {Mb}^{-1}$, and no instantaneous apoptosis was observed ([Hidema *et al.*, 2001](#_ENREF_11), [Hidema *et al.*, 1997](#_ENREF_14), [Hidema *et al.*, 2007](#_ENREF_15), [Iwamatsu *et al.*, 2008](#_ENREF_18), [Kang *et al.*, 1998](#_ENREF_20), [Quaite *et al.*, 1994](#_ENREF_35)). Based on the efficiency of protection and repair mechanisms, some bacterial species can recover from DNA damage induced-levels up to$400 CPD {Mb}^{-1}$ ([Zenoff *et al.*, 2006](#_ENREF_57)). Thus, we considered arbitrary $k_{s}=300 CPD {Mb}^{-1}$ and $k_{a}=500 CPD {Mb}^{-1}$ for both light and dark repair mechanisms (see Table 1).

Since photorepair of 6-4 photoproducts is 70% more efficient that CPD photorepair ([Chen *et al.*, 1994](#_ENREF_4), [Jiang *et al.*, 1997](#_ENREF_19)), and NER repair of 6-4PP is approximately 10-fold faster than NER repair of CPDs ([de Lima-Bessa *et al.*, 2008](#_ENREF_8), [Lo *et al.*, 2005](#_ENREF_27)), we adjusted the values accordingly (Table 1). Since the reviewed literature did not even hint at the $k_{s}$ (enzyme saturation point), and $k_{a}$ (level of DNA damage that causes instant cellular apoptosis) for 6-4PP repair, we considered the same values as for CPD repair. Parameters b_0_ and b_1_ were calculated for each r_max_, k_a_, and k_s_ combinations.

The model coefficients for the temperature dependence of the DNA damage induction and repair (Figure S4, see Table 1) were inferred from ([Li *et al.*, 2002](#_ENREF_25), [Takeuchi *et al.*, 1996](#_ENREF_46), [Waterworth *et al.*, 2002](#_ENREF_55)).


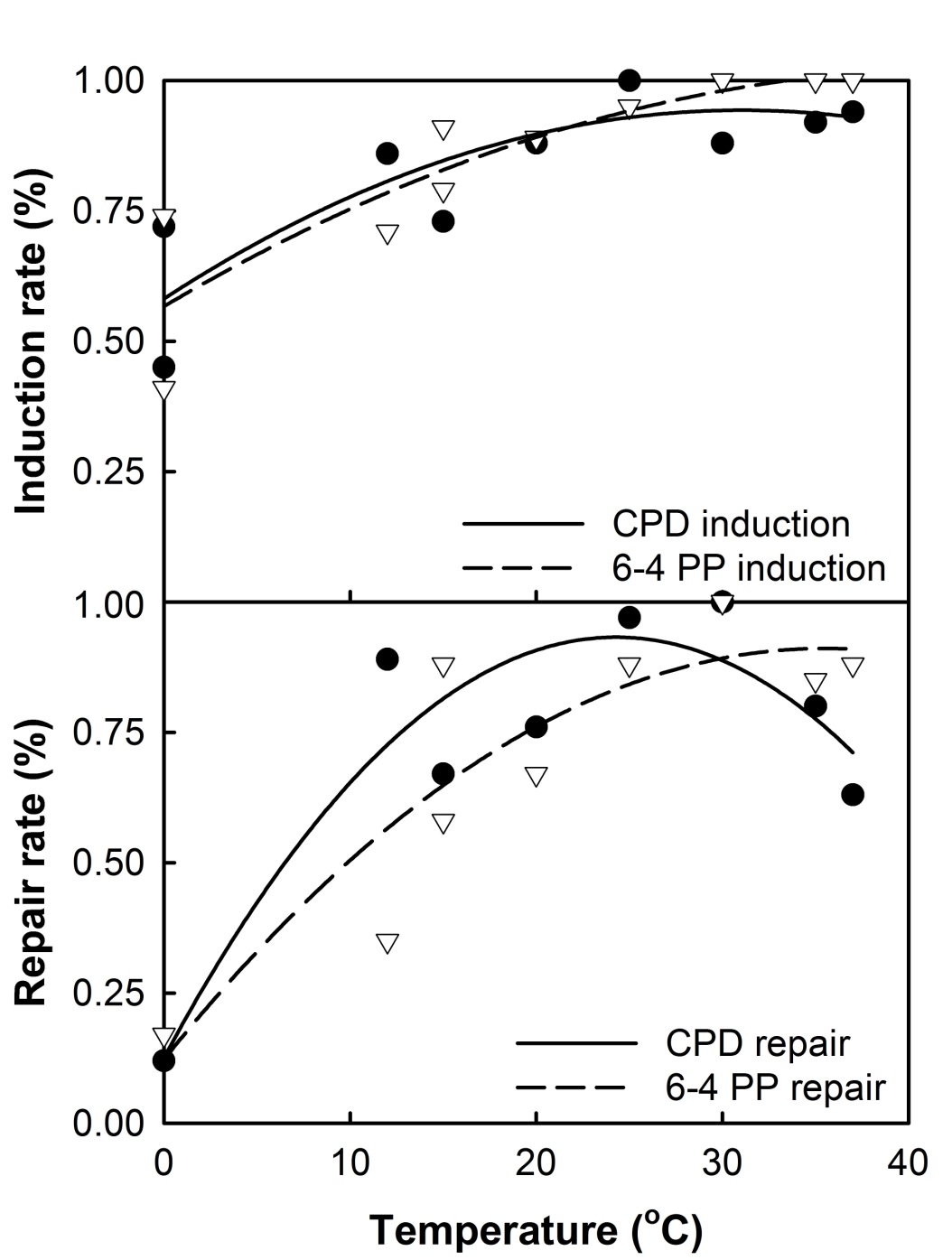


Figure S4: Temperature-dependent relative photoproducts induction and repair rates ([Li *et al.*, 2002](#_ENREF_25), [Takeuchi *et al.*, 1996](#_ENREF_46), [Waterworth *et al.*, 2002](#_ENREF_55)).

**2.4 Leaf expansion, longevity, and senescence**

Three leaf expansion parameters sets were considered: fast growing leaves (growth completed in seven days), medium growing leaves (growth completed in 15 days), and slow growing leaves (growth completed in 30 days). The corresponding estimates for the equation 13 parameters are presented in Table 1.

Published studies did not provide sufficient data for a quantitative relation between the levels of photoproducts and the percent of apoptotic cells (Figure S5).


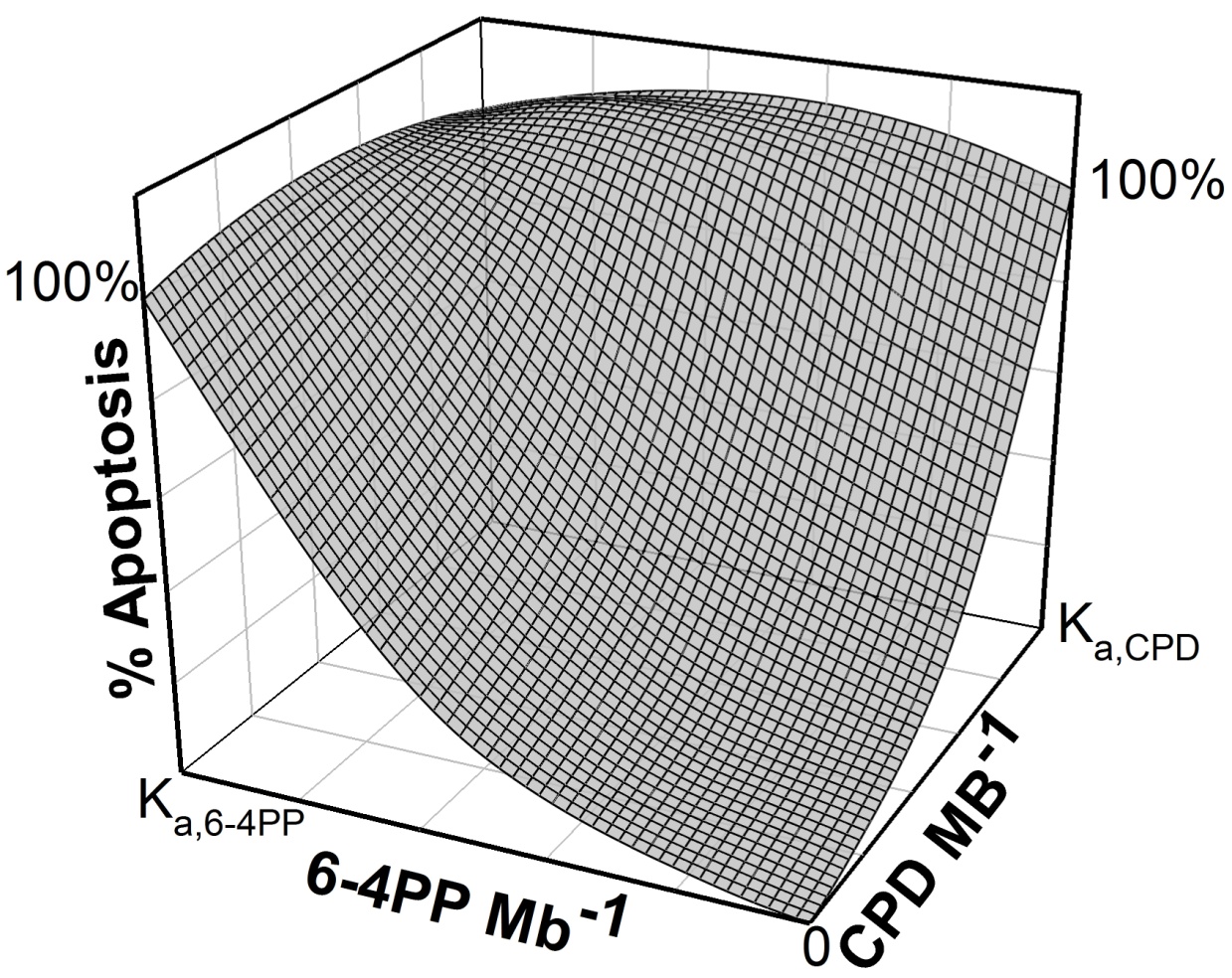


Figure 5S: Theoretical model of the percent of apoptotic cells as a function of CPD/6-4PPs Mb^-1^.

Instead, a linear equation inferred from Lo et al. ([Lo *et al.*, 2005](#_ENREF_27)) was used (see Table 1), with the warning that percent apoptosis predictions for CPD and 6-4PP levels above 55 CPD Mb^-1^ and 12 6-4PP Mb^-1^ are probably erroneous. To link the UV-B radiation induced DNA damage to leaf expansion, we used the following causal loop: if DNA damage is lower than 10 $CPD {Mb}^{-1}$, then cell division and cell expansion is unaffected; else if DNA damage is higher than 10 $CPD {Mb}^{-1}$, but lower than 500 $CPD {Mb}^{-1}$, cell division is delayed for 8-16 hours; if, after 8-16 hours, DNA damage is lower than 10 $CPD {Mb}^{-1}$, then cell division and cell expansion is resumed; if DNA damage is lower than 10 $CPD {Mb}^{-1}$ sooner then 8-16 hours, then cell division and cell expansion is resumed; if after 8-16 hours, DNA damage is higher than 10 $CPD {Mb}^{-1}$, or if the DNA damage is higher than 500 $CPD {Mb}^{-1}$, cells undergo apoptosis ([de Lima-Bessa *et al.*, 2008](#_ENREF_8), [Lo *et al.*, 2005](#_ENREF_27), [Zenoff *et al.*, 2006](#_ENREF_57)).

Although the leaf growth process is driven initially by active cell division, followed by, a decrease in the number of dividing cells, active cell expansion and differentiation, and leaf maturity ([Beemster *et al.*, 2005](#_ENREF_1)), the leaf expansion delays were not considered in this model. It has been showed, in both laboratory and field studies, that either processes, or either one, is responsible for leaf expansion inhibitions ([González *et al.*, 1998](#_ENREF_10), [Hofmann *et al.*, 2003](#_ENREF_16), [Hopkins *et al.*, 2002](#_ENREF_17), [Wargent *et al.*, 2009](#_ENREF_54)). Moreover, there are differences in the processes responsible for the cell expansion inhibitions for leaves from different locations on the same plant ([González *et al.*, 1998](#_ENREF_10)). Some of these studies are comparing no UV-B radiation treatments with ambient UV-B radiation treatments, or apply the supplemental UV-B radiation for only brief periods of time. It is possible that, similar to the pigment content, solar UV-B radiation might have a greater influence on the epidermal pigments content than the increased UV-B radiation ([Ryan *et al.*, 1998](#_ENREF_38), [Ryan *et al.*, 2002](#_ENREF_39)). We recognize that the photomorphogenic responses are important, and in some species may be the primary process leading the observed phenotypic plant responses to enhanced UV-B radiation. Since the rates for cell expansion inhibition are unclear at this time, all delays during the leaf growth were approximated by delays in cell division. This approximation may reduce the predictive power of the model.

**REFERENCES**

Beemster GT, De Veylder L, Vercruysse S *et al.* (2005) Genome-wide analysis of gene expression profiles associated with cell cycle transitions in growing organs of Arabidopsis. *Plant Physiology,* **138**, 734-743.

Bornman JF, Reuber S, Cen Y-O, Weissenbock G (1997) Ultraviolet radiation as a stress factor and the role of protective pigments. In: *Plants and UV-B: responses to environmental change.* (ed Lumsden PJ) pp Page. Cambridge, UK, Cambridge University Press.

Caldwell MM, Gold WG, Harris G, Ashurst CW (1983) A Modulated Lamp System for Solar Uv-B (280-320 Nm) - Supplementation Studies in the Field. *Photochemistry and Photobiology,* **37**, 479-485.

Chen JJ, Mitchell DL, Britt AB (1994) Light-Dependent Pathway for the Elimination of Uv-Induced Pyrimidine-(6-4) Pyrimidinone Photoproducts in Arabidopsis. *Plant Cell,* **6**, 1311-1317.

Day TA, Demchik SM (1996) Influence of enhanced UV-B radiation on biomass allocation and pigment concentrations in leaves and reproductive structures of greenhouse-grown Brassica rapa. *Vegetatio,* **127**, 109-116.

Day TA, Howells BW, Rice WJ (1994) Ultraviolet absorption and epidermal-transmittance spectra in foliage. *Physiologia Plantarum,* **92**, 207-218.

De La Rosa TM, Julkunen-Tiitto R, Lehto T, Aphalo PJ (2001) Secondary metabolites and nutrient concentrations in silver birch seedlings under five levels of daily UV-B exposure and two relative nutrient addition rates. *New Phytologist,* **150**, 121-131.

De Lima-Bessa KM, Armelini MG, Chigancas V, Jacysyn JF, Amarante-Mendes GP, Sarasin A, Menck CF (2008) CPDs and 6-4PPs play different roles in UV-induced cell death in normal and NER-deficient human cells. *DNA Repair (Amst),* **7**, 303-312.

Gausman HW, Rodriguez RR, Escobar DE (1975) Ultraviolet Radiation Reflectance, Transmittance, and Absorptance by Plant Leaf Epidermises1. *Agron. J.,* **67**, 720-724.

González R, Mepsted R, Wellburn AR, Paul ND (1998) Non-photosynthetic mechanisms of growth reduction in pea (Pisum sativum L.) exposed to UV-B radiation. *Plant, Cell & Environment,* **21**, 23-32.

Hidema J, I.-K. S, Sato T, Kumagai T (2001) Relationship between ultraviolet-B sensitivity and cyclobutane pyrimidine dimer photorepair in rice. *Journal of Radiation Research,* **42**, 295-303.

Hidema J, Kumagai T (1998) UV-B induced cyclobutil pyrimidine dimer and photorepair with progress of growth and leaf age in rice. *Journal of Photochemistry and Photobiology B: Biology,* **43**, 121-127.

Hidema J, Kumagai T, Sutherland BM (2000) UV radiation-sensitive Norin 1 rice contains defective cyclobutane pyrimidine dimer photolyase. *Plant Cell,* **12**, 1569-1578.

Hidema J, Kumagai T, Sutherland JC, Sutherland BM (1997) Ultraviolet B - sensitive rice cultivar deficient in cyclobutyl pyrimidine dimer repair. *Plant Physiology,* **113**, 39-44.

Hidema J, Taguchi T, Ono T, Teranishi M, Yamamoto K, Kumagai T (2007) Increase in CPD photolyase activity functions effectively to prevent growth inhibition caused by UVB radiation. *Plant Journal,* **50**, 70-79.

Hofmann RW, Campbell BD, Bloor SJ, Swinny EE, Markham KR, Ryan KG, Fountain DW (2003) Responses to UV-B radiation in Trifolium repens L. - physiological links to plant productivity and water availability. *Plant Cell and Environment,* **26**, 603-612.

Hopkins L, Bond MA, Tobin AK (2002) Ultraviolet-B radiation reduces the rates of cell division and elongation in the primary leaf of wheat (Triticum aestivum L. cv Maris Huntsman). *Plant, Cell and Environment,* **25**, 617-624.

Iwamatsu Y, Aoki C, Takahashi M *et al.* (2008) UVB sensitivity and cyclobutane pyrimidine dimer (CPD) photolyase genotypes in cultivated and wild rice species. *Photochem Photobiol Sci,* **7**, 311-320.

Jiang C-Z, Yee J, Mitchell DL, Britt AB (1997) Photorepair mutants of Arabidopsis. *Proceedings of the National Academy of Sciences,* **94**, 7441-7445.

Kang HS, Hidema J, Kumagai T (1998) Effects of light environment during culture on UV-induced cyclobutyl pyrimidine dimers and their photorepair in rice (Oryza sativa L.). *Photochemistry and Photobiology,* **68**, 71-77.

Kolb CA, Kaser MA, Kopecky J, Zotz G, Riederer M, Pfundel EE (2001) Effects of natural intensities of visible and ultraviolet radiation on epidermal ultraviolet screening and photosynthesis in grape leaves. *Plant Physiology,* **127**, 863-875.

Kon H, Ichibayashi R, Matsuoka N (2004) Changes of Diffuse UV-B Radiation on Clear Sky Days. *Journal of Agricultural Meteorology,* **60**, 285-290.

Lavola ANU, Julkunen-Tiitto R, Aphalo P, De La Rosa T, Lehto T (1997) The effect of u.v.-B radiation on u.v.-absorbing secondary metabolites in birch seedlings grown under simulated forest soil conditions. *New Phytologist,* **137**, 617-621.

Li J, Ou-Lee TM, Raba R, Amundson RG, Last RL (1993) Arabidopsis Flavonoid Mutants Are Hypersensitive to UV-B Irradiation. *The Plant Cell Online,* **5**, 171-179.

Li SS, Paulsson M, Bjorn LO (2002) Temperature-dependent formation and photorepair of DNA damage induced by UV-B radiation in suspension-cultured tobacco cells. *Journal of Photochemistry and Photobiology B-Biology,* **66**, 67-72.

Liu L, Gitz DC, Mcclure JW (1995) Effects of Uv-B on Flavonoids, Ferulic Acid, Growth and Photosynthesis in Barley Primary Leaves. *Physiologia Plantarum,* **93**, 725-733.

Lo HL, Nakajima S, Ma L, Walter B, Yasui A, Ethell DW, Owen LB (2005) Differential biologic effects of CPD and 6-4PP UV-induced DNA damage on the induction of apoptosis and cell-cycle arrest. *BMC Cancer,* **5**, 135.

Lodish H, Berk A, Kaiser CA *et al.* (2008) *Molecular Cell Biology,* New York, NY, W.H. Freeman and Company.

Meijkamp B, Aerts R, Van Der Staaij J, Tosserams M, Ernst W, Rozema J (1999) Effects of UV-B on secondary metabolites on plants. In: *Stratospheric Ozone Depletion: The Effects of Enhanced Uv-B Radiation on Terrestrial Ecosystems.* (ed Rozema J) pp Page. Leiden, The Netherlands, Backhuys Publishers.

Muller J, Behrens T, Diepenbrock W (2006) Use of a new sigmoid growth equation to estimate organ area indices from canopy area index in winter oilseed rape (Brassica napus L.). *Field Crops Research,* **96**, 279-295.

Noaa (2011) National Climatic Center. <http://www.ncdc.noaa.gov/>. Accessed May 2011. pp Page.

Nooden LD (2004) Introduction. In: *Plant Cell Death Processes.* (ed Nooden LD) pp Page. San Diego, CA, Academic Press.

Olsson LC, Veit M, Weissenbock G, Bornman JF (1998) Differential flavonoid response to enhanced UV-B radiation in Brassica napus. *Phytochemistry,* **49**, 1021-1028.

Qi Y, Bai S, Heisler GM (2003) Changes in ultraviolet-B and visible optical properties and absorbing pigment concentrations in pecan leaves during a growing season. *Agricultural and Forest Meteorology,* **120**, 229-240.

Quaite FE, Takayanagi S, Ruffini J, Sutherland JC, Sutherland BM (1994) DNA damage levels determine cyclobutil pyrimidine dimer repair mechanisms in alfalfa seedlings. *The Plant Cell,* **6**, 1635-1641.

Robberecht R, Caldwell MM (1978) Leaf Epidermal Transmittance of Ultraviolet-Radiation and Its Implications for Plant Sensitivity to Ultraviolet-Radiation Induced Injury. *Oecologia,* **32**, 277-287.

Robberecht R, Caldwell MM, Billings WD (1980) Leaf ultraviolet optical properties along a latitudinal gradient in the arctic-alpine life zone. *Ecology,* **61**, 612-619.

Ryan KG, Markham KR, Bloor SJ, Bradley JM, Mitchell KA, Jordan BR (1998) UVB radiation induced increase in quercetin: Kaempferol ratio in wild-type and transgenic lines of Petunia. *Photochemistry and Photobiology,* **68**, 323-330.

Ryan KG, Swinny EE, Markham KR, Winefield C (2002) Flavonoid gene expression and UV photoprotection in transgenic and mutant Petunia leaves. *Phytochemistry,* **59**, 23-32.

Sancar A (2003) Structure and Function of DNA Photolyase and Cryptochrome Blue-Light Photoreceptors. *Chemical Reviews,* **103**, 2203-2238.

Schmelzer E, Jahnen W, Hahlbrock K (1988) In situ localization of light-induced chalcone synthase mRNA, chalcone synthase, and flavonoid end products in epidermal cells of parsley leaves. *Proceedings of the National Academy of Sciences,* **85**, 2989-2993.

Setlow RB (1974) Wavelengths in Sunlight Effective in Producing Skin Cancer - Theoretical Analysis. *Proceedings of the National Academy of Sciences of the United States of America,* **71**, 3363-3366.

Sheahan JJ (1996) Sinapate esters provide greater UV-B attenuation than flavonoids in Arabidopsis thaliana (Brassicaceae). *American Journal of Botany,* **83**, 679-686.

Sisson WB (1981) Photosynthesis, Growth, and Ultraviolet Irradiance Absorbance of Cucurbita pepo L. Leaves Exposed to Ultraviolet-B Radiation (280-315 nm). *Plant Physiology,* **67**, 120-124.

Srivastava LM (2002) *Plant growth and development: hormones and environment,* San Diego, CA, Academic Press.

Takeuchi Y, Murakami M, Nakajima S, Kondo S, Nikaido O (1996) Induction and repair of damage to DNA in cucumber cotyledons irradiated with UV-B. *Plant Cell Physiology,* **37**, 181-187.

Taylor RM, Tobin AK, Bray CM (1997) DNA damage and repair in plants. In: *Plants and UV-B Responses to Environmental Change.* (ed Lumsden PJ) pp Page. Cambridge, UK, Cambridge University Press.

Tegelberg R, Veteli T, Aphalo PJ, Julkunen-Tiitto N (2003) Clonal differences in growth and phenolics of willows exposed to elevated ultraviolet-B radiation. *Basic and Applied Ecology,* **4**, 219-228.

Tevini M, Iwanzik W, Thoma U (1981) Some Effects of Enhanced Uv-B Irradiation on the Growth and Composition of Plants. *Planta,* **153**, 388-394.

Tevini M, Thoma U, Iwanzik W (1982) Effect of enhanced UV-B radiation on development and composition of plants. In: *Biological Effects of UV-B Radiation: Workshop : Papers.* (eds Bauer H, Caldwell MM, Tevini M, Worrest RC) pp Page. Munich, Germany, Gesellschaft fur Strahlen- und Umweltforschung.

Tevini M, Thoma U, Iwanzik W (1983) Effects of Enhanced Uv-B Radiation on Germination, Seedling Growth, Leaf Anatomy and Pigments of Some Crop Plants. *Zeitschrift Fur Pflanzenphysiologie,* **109**, 435-448.

Usda (2010) UV-B Monitoring and Research Program. <http://uvb.nrel.colostate.edu/UVB/>. Accessed January 2010. pp Page.

Vandestaaij JWM, Ernst WHO, Hakvoort HWJ, Rozema J (1995) Ultraviolet-B (280-320 Nm) Absorbing Pigments in the Leaves of Silene Vulgaris - Their Role in Uv-B Tolerance. *Journal of Plant Physiology,* **147**, 75-80.

Wargent JJ, Moore JP, Roland Ennos A, Paul ND (2009) Ultraviolet Radiation as a Limiting Factor in Leaf Expansion and Development. *Photochemistry and Photobiology,* **85**, 279-286.

Waterworth WM, Jiang O, West CE, Nikaido M, Bray CM (2002) Characterization of Arabidopsis photolyase enzymes and analysis of their role in protection from ultraviolet-B radiation. *Journal of Experimental Botany,* **53**, 1005-1015.

Yin XY, Goudriaan J, Lantinga EA, Vos J, Spiertz HJ (2003) A flexible sigmoid function of determinate growth. *Annals of Botany,* **91**, 361-371.

Zenoff VF, Sineriz F, Farias ME (2006) Diverse responses to UV-B radiation and repair mechanisms of bacteria isolated from high-altitude aquatic environments. *Applied and Environmental Microbiology,* **72**, 7857-7863.
